# Efficient Bulky Organo-Zinc Scorpionates for the Stereoselective Production of Poly(*rac*-lactide)s

**DOI:** 10.3390/polym13142356

**Published:** 2021-07-19

**Authors:** Marta Navarro, Andrés Garcés, Luis F. Sánchez-Barba, Felipe de la Cruz-Martínez, Juan Fernández-Baeza, Agustín Lara-Sánchez

**Affiliations:** 1 Departamento de Biología y Geología, Física y Química Inorgánica, Universidad Rey Juan Carlos, 28933 Móstoles, Spain; marta.navarro.sanz@urjc.es; 2 Departamento de Química Inorgánica, Orgánica y Bioquímica-Centro de Innovación en Química Avanzada (ORFEO-CINQA), Campus Universitario, Universidad de Castilla-La Mancha, 13071 Ciudad Real, Spain; Felipe.Cruz@uclm.es (F.d.l.C.-M.); Juan.FBaeza@uclm.es (J.F.-B.); Agustin.Lara@uclm.es (A.L.-S.)

**Keywords:** homogenous catalysis, zinc scorpionates, *rac*-lactide, heterotactic poly(*rac*-lactide)s

## Abstract

The direct reaction of the highly sterically demanding acetamidinate-based NNN′-scorpionate protioligand Hphbp^t^amd [Hphbp^t^amd = *N*,*N*′-di-p-tolylbis(3,5-di-tertbutylpyrazole-1-yl)acetamidine] with one equiv. of ZnMe_2_ proceeds in high yield to the mononuclear alkyl zinc complex [ZnMe(*κ*^3^-phbp^t^amd)] (**1**). Alternatively, the treatment of the corresponding lithium precursor [Li(phbp^t^amd)(THF)] with ZnCl_2_ yielded the halide complex [ZnCl(*κ*^3^-phbptamd)] (**2**). The X-ray crystal structure of **1** confirmed unambiguously a mononuclear entity in these complexes, with the zinc centre arranged with a pseudotetrahedral environment and the scorpionate ligand in a *κ*^3^-coordination mode. Interestingly, the inexpensive, low-toxic and easily prepared complexes **1** and **2** resulted in highly efficient catalysts for the ring-opening polymerisation of lactides, a sustainable bio-resourced process industrially demanded. Thus, complex **1** behaved as a single-component robust initiator for the living and immortal ROP of *rac*-lactide under very mild conditions after a few hours, reaching a TOF value up to 5520 h^−1^ under bulk conditions. Preliminary kinetic studies revealed apparent zero-order dependence on monomer concentration in the absence of a cocatalyst. The PLA materials produced exhibited narrow dispersity values, good agreement between the experimental *M*_n_ values and monomer/benzyl alcohol ratios, as well as enhanced levels of heteroselectivity, reaching *P*_s_ values up to 0.74.

## 1. Introduction

The rational use of natural resources and the efficient management of waste materials represent two of the most important challenges in this century [1,2] for the sustainability of our planet, in accordance with the “Twelve Principles of Green Chemistry” [3].

**Chart 1:**
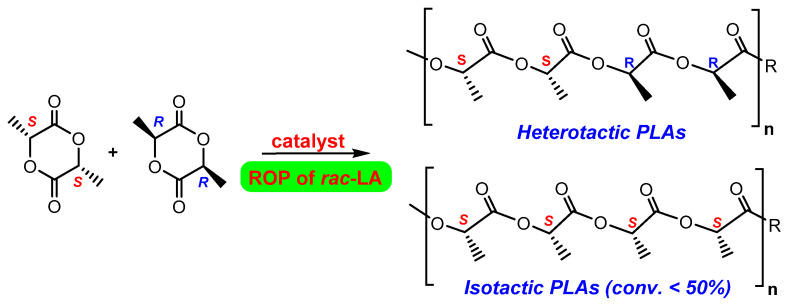
Preparation of hetero- and isotactic poly(lactide)s via the ROP of *rac*-latide mediated by steroselective metal-based catalysts.

Polylactide (PLA) [4,5,6] is an annually biorenewable material that has attracted great attention [7,8,9,10,11,12,13,14] as a result of the important concerns about the depletion of fossil-fuel feedstocks along with the environment waste-derived problems. PLAs can be obtained through the ring-opening polymerisation (ROP) of the biosourced cyclic ester of lactide (LA), by employing efficient metal-based initiators. This process offers excellent control of molecular weight, molecular-weight distribution and stereoselectivity in the growing polymer chains (see Chart 1). Particularly, the production of this top commercial material is annually increasing, and it now represents close to 7% of the total bioplastics produced worldwide [15], since PLAs find multiple biomedical and pharmaceutical applications, including the controlled release of drugs [16,17], regenerative medicine [18] and wound healing [19], as well as in packaging and agriculture as a real alternative to conventional commodity thermoplastics [20,21].

**Chart 2:**
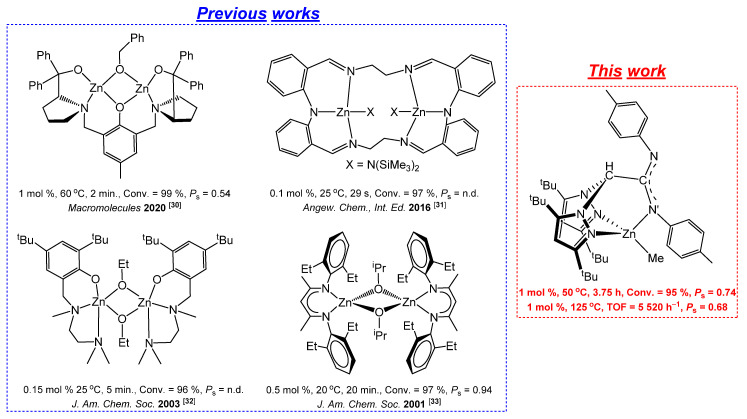
Representative achiral zinc-based catalysts for the synthesis of hetero-enriched poly(*rac*-lactide)s.

In this context, biologically benign metal-based catalysts are of great interest for the production of this bioassimilable material, with calcium [22,23,24,25], magnesium [26,27,28,29] and zinc [30,31,32,33,34,35] (see Chart 2), as the most representative centres, although other metals from group 13 (aluminium [36] and indium [37]), group 4 [38], as well as rare earth [39] metals, have been also successfully described, all of them supported by a wide variety of ancillary ligands. These examples constitute a much greener alternative to the toxic industrially employed tin(II) 2-ethylhexanoate, which, despite its robustness, offers poor control of polymer parameters [40].

Particularly, our research group has successfully reported over the last few years a series of well-defined mono- and multinuclear organo-zinc [41,42,43,44,45,46] scorpionate complexes as efficient one-component initiators for the living ROP of cyclic esters [47], and significant levels of isotacticity (*P*_i_ = 0.77 [44,45]–0.88 [41]) have been successfully attained.

On the bases of our previous expertise [41,42,43,44,45,46], now we endeavour to develop a series of inexpensive, low-toxic and easy-to-prepare zinc-based catalysts that are very efficient and selective in this industrially-demanded process. For this propose, a very successful sterically hindered acetamidinate-based NNN’-scorpionate [48] from our extended ligand library [48,49,50] has been employed.

Hereby the preparation of novel mononuclear zinc complexes supported by a sterically hindered scorpionate is reported. These complexes behave as single-component initiators for the living and immortal ROP of *rac*-lactide to produce steroselectively poly(*rac*-lactide)s with enhanced degrees of heterotacticity.

## 2. Materials and Methods

### 2.1. Materials

All manipulations were carried out under a nitrogen atmosphere using standard Schlenk techniques or a glovebox. Solvents were predried over sodium wire and distilled under nitrogen from sodium (toluene and *n*-hexane) or sodium-benzophenone (THF and diethyl ether). Deuterated solvents were stored over activated 4 Å molecular sieves and degassed by several freeze–thaw cycles. The protioligand Hphbptamd was prepared according to the procedures in the literature [48]. ZnMe_2_ (Sigma-Aldrich, Munich, Germany) was used as purchased and ZnCl_2_ (Sigma-Aldrich, Munich, Germany) was predried by several heat toluene suspension-vacuo cycles before use. *rac*-lactide (Sigma-Aldrich, Munich, Germany) was sublimed twice, recrystallised from THF, and finally sublimed again prior to use.

### 2.2. Experimental

#### 2.2.1. Nuclear Magnetic Resonance Spectroscopy (NMR)

The NMR spectra of complexes were recorded on a Varian Inova FT-500 spectrometer and were referenced to the residual deuterated solvent signal. ^1^H NMR homodecoupled and NOESY-1D spectra were recorded on the same instrument with the following acquisition parameters: irradiation time 2 s and 256 scans, using standard VARIAN-FT software. Furthermore, 2D NMR spectra were acquired using the same software and processed using an IPC-Sun computer.

The microstructures of PLA samples were determined by examination of the methine region in the homodecoupled ^1^H NMR spectrum of the polymers recorded at room temperature in CDCl_3_ with concentrations in the range 1.0 to 2.0 mg/mL.

#### 2.2.2. Elemental Analysis

Microanalyses were performed with a Perkin-Elmer 2400 CHN analyser (Perkin Elmer, Inc., Waltham, MA, USA).

#### 2.2.3. Gel Permeation Chromatography (GPC)

The molecular weights (*M*_n_) and the molecular-mass distributions (*M*_w_/*M*_n_) of polymer samples were measured by gel permeation chromatography (GPC) performed on a Shimadzu LC-20AD GPC equipped with a TSK-GEL G3000Hxl column and an ELSD-LTII light-scattering detector (Shimadzu Corporation, Kyoto, Japan). The GPC column was eluted with THF at 40 °C at 1 mL/min and was calibrated using eight monodisperse polystyrene standards in the range 580–483,000 Da.

#### 2.2.4. MALDI-TOF Mass Spectrometry

MALDI-ToF MS data were acquired with a Bruker ULTRAFLEX III ToF/ToF spectrometer (Bruker, Billerica, MA, USA), using a NdYAG laser source (355 nm) in reflector mode with a positive acceleration voltage of 25 kV. Samples were prepared as follows: PLA was dissolved in dichloromethane 1.5 mg/mL and mixed with matrix (DCTB 10 mg/mL in dichloromethane) and NaI (2 mg/mL in acetone) in a 20:5:0.5 ratio (matrix:sample:NaI). Before evaporation, 0.5 mL of the mixture solution was deposited on the sample plate. External calibration was performed using Peptide Calibration Standard II + ACTH clip 7–38, ACTH clip 1–39 + INSULINE (covered mass range: 1000–7000 Da).

#### 2.2.5. Crystallographic Refinement and Structure Solution

Crystals suitable for X-ray diffraction were obtained for **1**. The crystals were selected under oil and attached to the tip of a nylon loop. The crystals were mounted in a stream of cold nitrogen at 240–250 K and centred in the X-ray beam. A single crystal of 1 was measured at 100 K with a Bruker Kappa Apex II system, with graphite-monochromated Mo Kα radiation (λ = 0.71073 Å) from conventional sealed tubes. The initial cell constants were obtained from three series of scans at different starting angles. The reflections were successfully indexed by an automated indexing routine built into the SAINT program [51]. The absorption correction was based on fitting a function to the empirical transmission surface as sampled by multiple equivalent measurements [52]. A successful solution using the direct methods [53] provided most non-hydrogen atoms from the E-map. The remaining non-hydrogen atoms were located in an alternating series of least-squares cycles and difference Fourier maps. All non-hydrogen atoms were refined with anisotropic displacement coefficients unless specified otherwise. All hydrogen atoms were included in the structure factor calculation at idealised positions and were allowed to ride on the neighbouring atoms with relative isotropic displacement coefficients.

Final R(F), wR(F2), and goodness-of-fit agreement factors, details on the data collection, and analysis for **1** can be found in Table S1 in the Supporting Information.

### 2.3. General Procedures

#### 2.3.1. Preparation of Compounds **1**–**2**

*Synthesis of [ZnMe(κ^3^-phbp^t^amd)]* (**1**). In a 100 mL Schlenk tube, pbp^t^amd-H (1.00 g, 1.68 mmol) was dissolved in dry *n*-hexane (25 mL) and cooled to −70 °C. A solution of ZnMe_2_ (1.2 M in toluene) (1.40 mL, 1.68 mmol) was added and the mixture was allowed to warm up to room temperature and stirred for 2 h. After concentration and being cooled at −26 °C, compound **1** was obtained as colorless crystals. Yield: 0.91 g, 80%. Anal. Calcd. for C_39_H_56_N_6_Zn: C, 69.47; H, 8.37; N, 12.46. Found: C, 69.50; H, 8.39; N, 12,51. ^1^H-NMR (C_6_D_6_, 297 K), δ 6.37 (s, 1 H, CH), 6.15 (d, ^3^J_H-H_ = 8.2 Hz, 2 H, Ar-H), 6.03 (d, ^3^J_H-H_ = 8.2 Hz, 2 H, Ar-H), 5.98 (d, ^3^J_H-H_ = 8.2 Hz, 4 H, Ar-H), 5.24 (s, 2 H, H^4,4′^), 1.21(s, 3 H, N′C_6_H_4_*Me*), 1.17 (s, 3 H, NC_6_H_4_*Me*), 0.61 (s, 18 H, ^t^Bu^5,5′^), 0.59 (s, 18 H, ^t^Bu^3,3′^), −0.53 (s, 3 H, ZnMe). ^13^C-{^1^H}-NMR (C_6_D_6_, 297 K), δ 162.9 (C^b^), 155.1, 154.9 (C^3,3′or5,5′^), 152.1–119.9 (N*C*_6_H_4_Me), 102.8 (C^4,4′^), 77.0 (C^a^), 32.5 (^t^Bu^3^), 32.3 (^t^Bu^3′^), 31.1 (^t^Bu^5^), 30.3 (^t^Bu^5′^), 20.9 (N′C_6_H_4_*Me*), 20.7 (NC_6_H_4_*Me*), −6.94 (ZnMe).

*Synthesis of [ZnCl(κ^3^-phbp^t^amd)]* (**2**). In a 100 mL Schlenk tube, Hpbp^t^amd (1.00 g, 1.68 mmol) was dissolved in dry tetrahydrofuran (25 mL) and cooled to −70 °C. A solution of *n*-BuLi (2,5 M in hexane) (0.67 mL, 1.68 mmol) was added to the mixture, and it was allowed to warm up to room temperature and stirred for 30 min. A solution of ZnCl_2_ (0.23 g, 1.68 mmol) in dry tetrahydrofuran (25 mL) was added dropwise to the previous cooled mixture, and the reaction mixture was stirred for 2 h. The solvent was removed in vacuo and extracted with toluene (25 mL), and the resulting solution was concentrated ca. 10 mL and was cooled at −26 °C to give compound **2** as a white semicrystalline solid. Yield: 0.94 g, 80%. Anal. Calcd. for C_38_H_53_ClN_6_Zn: C, 65.70; H, 7.69; N, 12.10. Found: C, 65.81; H, 7.75; N, 12.15. ^1^H-NMR (CDCl_3_, 297 K), δ 7.66 (s, 1 H, CH), 6.53 (d, ^3^J_H-H_ = 8 Hz, 6 H, Ar-H), 6.24 (d, ^3^J_H-H_ = 8,2 Hz, 2 H, Ar-H), 6.08 (s, 2H, H^4,4′^), 2.04 (s, 3 H, N′C_6_H_4_*Me*), 2.01 (s, 3 H, NC_6_H_4_*Me*), 1.56 (s, 18 H, ^t^Bu^5,5′^), 1.43 (s, 18 H, ^t^Bu^3,3′^). ^13^C-{^1^H}-NMR (CDCl_3_, 297 K), δ 164.4 (C^b^), 155.9, 154.4 (C^3,3′or5,5′^), 147.6–121.9 (N*C*_6_H_4_Me), 103.4 (C^4,4′^), 77.1 (C^a^), 32.6 (^t^Bu^3^), 32.5 (^t^Bu^3′^), 31.05 (^t^Bu^5^), 30.30 (^t^Bu^5′^), 20.8 (N′C_6_H_4_*Me*), 20.7 (NC_6_H_4_*Me*).

#### 2.3.2. Typical Polymerisation Procedures

The polymerisation of *rac*-lactide (LA) was performed on a Schlenk line in a flame-dried Schlenk tube equipped with a magnetic stirrer. The Schlenk tubes were charged in a glovebox with the required amount of LA and initiator, separately, and then attached to the vacuum line. The initiator and LA were dissolved in the appropriate amount of solvent and temperature equilibration was ensured in both Schlenk tubes by stirring the solutions for 15 min in a bath. Under immortal conditions, the corresponding equiv of BnOH (benzyl alcohol as cocatalyst, stock solution) were also included in the LA Schlenk flask solution. Next, the appropriate amount of initiator was added by using a syringe, and polymerisation times were measured from that point. Polymerisations were stopped by injecting a solution of acetic acid in water (0.35 M). Polymers were precipitated in methanol, filtered off, redissolved, and reprecipitated in methanol, and dried in vacuo to a constant weight. All kinetics experiments were repeated at least twice and were mutually consistent.

## 3. Results

### 3.1. Synthesis and Characterisation of NNN’-Scorpionate Alkyl and Chloride Zinc Complexes ***1*** and ***2***

**Scheme 1:**
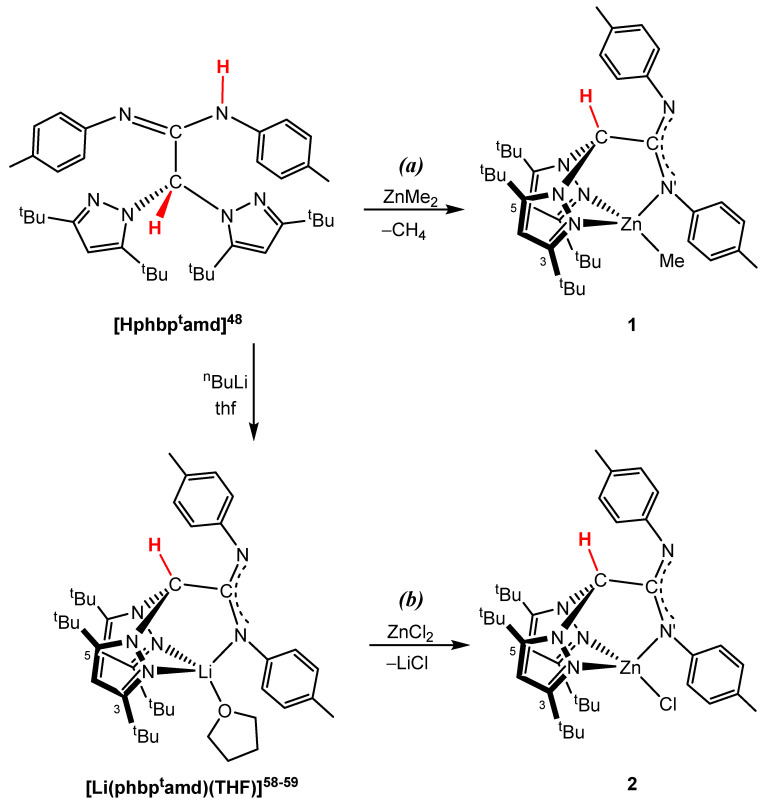
Preparation of the acetamidinate-based NNN′-scorpionate zinc complexes **1** and **2**.

The high sterically demanding acetamidinate-based scorpionate protioligand Hphbp^t^amd [48] [Hphbp^t^amd = *N*,*N*′-di-p-tolylbis(3,5-di-tertbutylpyrazole-1-yl)acetamidine] was initially reacted with one equivalent of ZnMe_2_ in toluene at room temperature. This reaction cleanly afforded the mononuclear amidinate-based scorpionate zinc complex [ZnMe(κ^3^-phbp^t^amd)] **1**, a white solid in good yield (ca. 80%) (see Scheme 1a).

Alternatively, we considered it very interesting to prepare a halide derivative analog to **1**, since diverse M^II^-halide species have been also described as efficient catalysts for the ROP of lactides [54,55,56,57]. Thus, the treatment of the protioligand Hphbp^t^amd with ^n^BuLi in thf at 0 °C afforded the scorpionate lithium salt [Li(phbp^t^amd)(THF)] [58] [phbp^t^amd = N,N’-di-p-tolylbis(3,5-di-tert-butylpyrazole-1-yl)acetamidinate], and subsequent reaction with ZnCl_2_ yielded the halide complex [ZnCl(κ^3^-phbp^t^amd)] **2**, a yellow pail solid in good yield (ca. 80%) (see Scheme 1b). Compounds **1** and **2** are stable to the air and moisture for 5 h and 24 h, respectively, but complex **1** readily decomposed when dissolved in dichloromethane, possibly through a protonolysis reaction.

Interestingly, attempts to activate the bridging C–H group by the addition to complex **1** of a second equivalent of ZnMe_2_ to prepare potentially more active bimetallic catalysts through an intramolecular cooperative mechanism were fruitless, in contrast to the behaviour previously observed in our group for the preparation of homobimetallic complexes containing other metals and bearing lower sterically demanded scorpionates [58,59].

**Figure 1:**
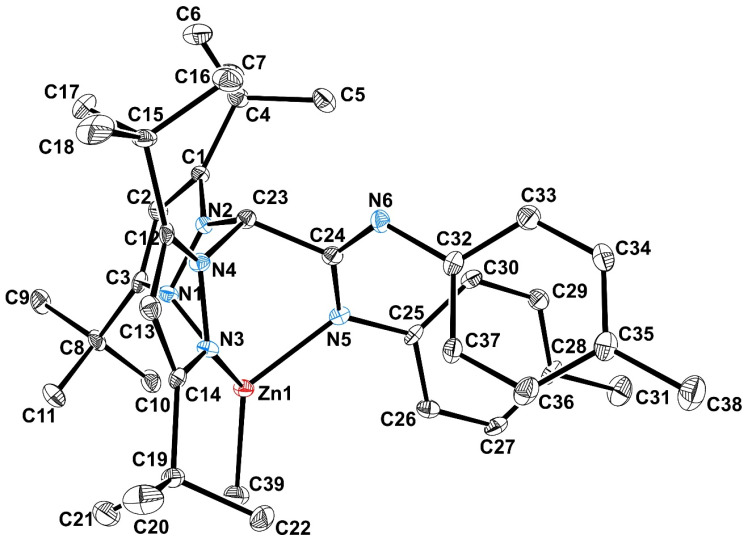
ORTEP view of [ZnMe(*κ*^3^-phbp^t^amd)] **1**. Hydrogen atoms have been omitted for clarity. Thermal ellipsoids are drawn at the 30% probability level.

The ^1^H and ^13^C {^1^H} NMR spectra of **1** and **2** in benzene-d_6_ at room temperature show similar patterns. The spectra show one sets of resonances for the ^t^Bu^3,5^ and H^4^ in the sterically hindered pyrazole rings, one signal for the CH group and two different signals for the acetamidinate fragment, which are in agreement with a monodentate binding for the acetamidinate moiety (see Scheme 1). Additionally, one signal at negative chemical shift appears for the Zn-alkyl group in complex **1** (see Figures S1 and S2 in the SI). In addition, the signals for C^4^, ^t^Bu^3,5^ in the pyrazole rings, as well as for the 4-MePh amidinate substituents were assigned by ^1^H−^13^C heteronuclear correlations (g-HSQC). The proposed structures for the acetamidinate complexes **1** and **2** were further confirmed by the X-ray molecular analysis (see below Figure 1).

Single crystals of complex **1** suitable for X-ray diffraction were easily grown from a toluene solution at –26 °C. The molecular structure is depicted in Figure 1. Selected bond lengths and angles are collected in Table 1, and the crystallographic details are reported in Table S1 in the SI. The molecular structure of **1** consists of a monomeric arrangement in the solid state. The zinc metal exhibits a distorted tetrahedral geometry, with the scorpionate ligand in a κ^3^-NNN′ coordination mode. The N(1)–Zn and N(3)–Zn bond lengths [2.167(5) Å and 2.135(5) Å] are well balanced and compare well with that observed in the analog acetamidinate-based scorpionate zinc alkyls [48] but are considerably longer than the N(5)–Zn bond length [2.034(5) Å]. The solid-state structure also confirms that the acetamidinate is coordinated in a monodentate fashion with the Zn atom, and delocalisation is also evidenced in the N–C–N moiety of the acetamidinate, with the bond lengths C(24)–N(5) and C(24)–N(6) ranging from 1.351(8) Å to 1.295(8) Å. An analog crystal structure of complex **2** has been previously reported by our group [48].

### 3.2. Catalytic Studies on the ROP of rac-LA for the Production of Poly(rac-lactide) ***3***

These first studies were aimed at comparing the activity and stereoselectivity of these new mononuclear high sterically hindered acetamidinate-based initiators **1** and **2** with analog scorpionate zinc alkyls [41,42,43,44,45,46] and other remarkable dinuclear [30,31,32,33,34,35] and mononuclear [60,61,62,63,64] organo-zinc initiators reported to date in the ROP of *rac*-LA.

**Scheme 2:**
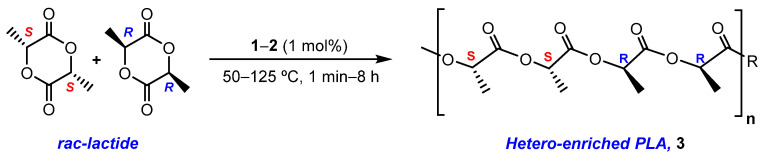
ROP of *rac*-lactide for the production of poly(*rac*-lactide) **3** catalysed by complexes [ZnMe(*κ*^3^-phbp^t^amd)] **1** and [ZnCl(*κ*^3^-phbp^t^amd)] **2**.

Thus, complexes **1** and **2** were systematically assessed in the ring-opening polymerisation (ROP) of the polar monomer *rac*-lactide (*rac*-LA) at 40–60 °C in tetrahydrofuran, toluene, and dichoromethane as solvents and, in bulk conditions (125 °C), under a nitrogen atmosphere for the production of poly(*rac*-lactide) (PLA) **3** (see Scheme 2 and Table 2). The experimental medium-low M_n_ values of the PLAs produced were determined by size exclusion chromatography (SEC) using the Mark–Houwink corrections [65,66] and showed good agreement with the expected theoretical calculated values considering one growing polymer chain per zinc centre (see Table 2). In addition, examination of the resulting polyesters revealed a monomodal weight distribution, with narrow dispersity values ranging from 1.05 to 1.35 (see Figure S3 in the SI).

Complexes **1** and **2** were initially evaluated in the polymerisation of *rac*-LA employing 100 equiv. of monomer at mild conditions to demonstrate their catalytic activity. Thus, the alkyl complex **1** behaved as a very active single-component living initiator, reaching almost complete conversion (95%) in tetrahydrofuran in less than 4 h at 50 °C (see Table 2, entry 4). Interestingly, the halide complex **2** transformed 52% of the initial monomer in 6 h at 60 °C (see Table 2, entry 5), whilst analog halide zinc-based initiators have been described to operate in this process under much more harsher conditions (i.e., 150 °C [55] and 130 °C [57]) and have been reported to need up to 5 days at room temperature [56] to reach high conversions of **3**.

It is worth noting that the presence of this high sterically hindered ligand that additionally incorporates phenyl substituents in the acetamidinate fragment very efficiently prevents the possible formation of the homoleptic six-coordinate sandwich-like [Zn(phbp^t^amd)_2_] that disfavours catalytic performance, as previously observed for low sterically hindered zinc-based scorpionate analogs [67].

Interestingly, catalyst **1** presents an induction period, since limited catalytic activity was observed during the first 2 h (Table 2, entries 1 and 2), similarly to the bis(imino)diphenylamido zinc-based catalyst previously reported by Williams et al. (Chart 2 [31]) and other zinc-based catalysts [34,35] This induction period is possible due to the delay in the formation of the essential catalyst active species in the monomer pool, which are highly influenced by both the steric and electronic effects of the scorpionate ligand. Similarly to this previous work, preliminary kinetic studies on catalyst **1** revealed zero dependence to the monomer concentration, which means that the transformation of the monomer with time remains constant (see Figure S4 in the SI). Very interestingly, in the presence of an excess of benzyl alcohol (5 equiv) as a cocatalyst, **1** efficiently mediated the immortal ROP of this monomer at 50 °C, as evidenced by the very narrow dispersity value and the good agreement between the experimental M_n_ values and monomer/benzyl alcohol ratios (see Table 2, entry 6). Under this immortal behaviour, 60% of the monomer was transformed in only 1 h and no induction period was observed.

Moreover, the well-controlled living performance of **1** was confirmed through a double-feed experiment, resulting in a polymer chain extension of **3** with comparable polymer features, which confirms the existence of a single type of reaction site (see Table 2, entries 4 and 7).

The effect of temperature and solvent was also examined. Thus, initiator **1** dramatically reduced conversion level at 40 °C, and only traces of **3** were observed after 24 h of reaction. In addition, a significant reduction in catalytic performance was observed on using toluene as solvent, reaching a poor 19% of conversion after 8 h at 50 °C, possibly as a result of the complexation of the zinc ions when using the tetrahydrofuran coordinating solvent, thus leading to an increase in the nucleophilicity of the alkyl initiating group and the alkoxide propagating chains (Table 2, entries 8 and 9). Not unexpectedly, initiator **2** did not transform any monomer either in toluene at 60–90 °C and in dichloromethane at 40 °C, after 48 h of reaction in both cases.

Very interestingly, complex **1** displayed near-complete conversion of 100 equiv of *rac*-LA into **3** under bulk conditions (125 °C) after just 1 min, reaching a TOF value of 5520 h^−1^, whilst complex **2** needed 2 min to transform 59% of the monomer (TOF = 1770 h^−1^). In view of these promising results, we were encouraged to carried out a further experiment employing partially purified (twice-sublimed) monomer, which confirmed the lower moisture sensitivity of complex **1** in comparison with analog zinc(II)-based scorpionate initiators [42,46,67], reaching 42% of conversion in 5 min (TOF = 504 h^−1^) (see Table 2, entries 10–12, respectively).

This initiator offered activities much higher than mononuclear sterically demanded NNN’ amidinate-based zinc scorpionate analogs [67] and similar activity to the NNO alkoxide-based zinc scorpionate counterparts previously described in our group [46]. However, complex **1** needs more temperature to initiate efficiently the ROP of *rac*-LA than the recently reported amine-functionalised NNO-scorpionate analogs [42] (Table 2, entries 13–15, respectively). Moreover, these activity values had lower results than the dinuclear species described above (Chart 2, [30,31,32,33]) or the mononuclear complexes recently reported by Ma employing zinc complexes bearing benzoimidazolyl-[60], pyridyl-[61] or [NNNO]-type tetradentate-[62] based aminophenolate ligands, which efficiently operate at room temperature for a few minutes; however, in the last case, the presence of ^i^PrOH as a cocatalyst was necessary [61,62].

In addition, low-molecular-weight materials of **3** prepared with initiator **1** were inspected by MALDI−ToF MS (see Figure S5 in the SI). Moreover, end-group analyses by ^1^H NMR of poly(*rac*-lactide) oligomers were also examined (see Figure S6 in the SI). These two results provide evidence that the ring-opening of *rac*-LA occurs by the initial addition of the alkyl fragment to the monomer in the PLA materials, with the cleavage of the acyl-oxygen bond [69] followed by further monomer additions to the (macro)alcohols.

More importantly, ^1^H-NMR microstructure analysis in the poly(*rac*-lactide) **3** produced in tetrahydrofuran revealed enhanced levels of heteroactivity imparted by **1**, reaching a P_s_ of 0.74, probably through a chain end control mechanism [59] (Table 2, entry 4, see Figure S7 in the SI).

Very interestingly, this value is close to the highest data previously reported for the heterotacticity displayed by zinc-based scorpionate initiators prepared in our group in the steroselective ROP of *rac*-LA [41,42,43,44,45,46,50,67] For instance, complex 1 exerted much higher heteroselectivity than the amine-functionalised NNO-scorpionate zinc analogs [42] (*P*_s_ = 0.62) than the sterically demanded NNN’ amidinate-based zinc scorpionates (*P*_s_ = 0.68) [67], close to the value reported for the NNO alkoxide-based zinc scorpionates (*P*_s_ = 0.77) [46] (Table 2, entries 13–15, respectively). Moreover, this value has results that were significantly higher than those reported for racemic pyridyl- (*P*_s_ = 0.49) [61] and [NNNO]-type tetradentate (*P*_s_ = 0.54–0.60)-based [62] aminophenolate zinc complexes. This is certainly attributed to the more sterically demanding environment produced by all bulkier substituents in the phbp^t^amd scorpionate ligand. Furthermore, under bulk conditions, initiator 1 exerted moderate values of heterotacticity (*P*_s_ = 0.68) (Table 2, entry 10, see Figure S8 in the SI).

## 4. Conclusions

Hereby, the easy preparation of zinc-based complexes [ZnR(κ^3^-phbptamd)] (R = Me, Cl) supported by a high sterically hindered NNN’-acetamidinate scorpionate is described. X-ray diffraction analysis for [ZnMe(κ^3^-phbptamd)] evidenced a mononuclear species and the zinc centre in a pseudotetrahedral disposition with the scorpionate ligand in a κ^3^-fashion.

Very interestingly, these complexes behaved as highly efficient catalysts for the ROP of *rac*-lactide. Thus, **1** can act as an effective and robust single-component initiator for the living ROP of *rac*-LA under very mild conditions with a 2 h induction period, as shown by the narrow dispersity values of the PLAs prepared. As evidence, this initiator is capable of reaching a TOF value up to 5520 h^−1^ under bulk conditions. Preliminary kinetic studies confirmed apparent zero-order dependence on monomer concentration in the absence of a cocatalyst, whereas in the presence of HOBn, catalyst **1** displayed an immortal performance, with nice agreement between the M_n_ observed and monomer/benzyl alcohol ratios. More importantly, the degree of steric hindrance of the scorpionate ligand in **1** exerts enhanced levels of hetero-activity during polymerisation to produce steroselective poly(*rac*-lactide)s, reaching a P_s_ value of 0.74.

We consider these results to represent an important further step forward in the search of inexpensive, low-toxic, and easy-to-prepare metal-based catalysts that are interesting for industrial applicability and which are capable of operating efficiently in the sustainable bioresourced ROP of lactides.

## Figures and Tables

**Table 1:** Selected bond lengths (Å) and angles (°) for **1**.

Distances (Å)		Angles (°)	
N(1)-Zn(1)	2.167 (5)	C(39)-Zn(1)-N(5)	128.2 (2)
N(3)-Zn(1)	2.135 (5)	C(39)-Zn(1)-N(3)	125.8 (2)
N(5)-Zn(1)	2.034 (5)	N(5)-Zn(1)-N(3)	90.8 (2)
C(39)-Zn(1)	1.970 (7)	C(39)-Zn(1)-N(1)	124.2 (2)
C(24)-N(6)	1.295 (8)	N(5)-Zn(1)-N(1)	91.76 (18)
C(24)-N(5)	1.351 (8)	N(3)-Zn(1)-N(1)	83.00 (18)
C(23)-C(24)	1.540 (8)	N(6)-C(24)-N(5)	135.5 (6)
		N(6)-C(24)-C(23)	110.3 (5)
		N(5)-C(24)-C(23)	114.1 (5)
		N(2)-C(23)-N(4)	110.6 (4)
		N(2)-C(23)-C(24)	114.9 (5)
		N(4)-C(23)-C(24)	106.7 (4)

**Table 2:** Polymerisation of *rac*-lactide catalysed by initiators **1** and **2** ^a^.

Entry	Initiator	Temp (°C)	Time (h)	Yield (g)	Conv (%) ^b^	*M*_n(theor.)_ (Da) ^c^	*M*_n_ (Da) ^d^	*M*_w_/*M*_n_^d^	*P*_s_^e^
1	**1**	50	1	0.17	13	1900	2500	1.05	0.74
2	**1**	50	2	0.35	27	3900	4800	1.09	0.73
3	**1**	50	3	0.90	70	10,100	10,400	1.12	0.74
4	**1**	50	3.75	1.23	95	13,700	13,000	1.13	0.74
5	**2**	60	6	0.67	52	7500	7200	1.12	0.72
6	**1 + HOBn** (1:5)	50	1	0.78	60	1700	1900	1.08	ND ^k^
7	**1** ^f^	50	7.5	2.41	93	26,800	25,200	1.12	ND
8	**1**	40	24	—	traces	—	—	—	—
9	**1** ^g^	50	8	0.25	19	2700	3100	1.06	ND
10	**1** ^h^	125	1 min	1.19	92	13,200	12,600	1.28	0.68
11	**2** ^h^	125	2 min	0.76	59	8500	9100	1.29	0.67
12	**1** ^i^	125	5 min	0.54	42	6000	6400	1.35	—
13	**[Zn(Et)(tbptamd)] ^j^**, [67]	110	48	0.81	63	9100	8700	1.11	0.68
14	**[Zn(Me)(bpzampe)] ^j^**, [42]	20	4	1.10	85	12,200	12,300	1.15	0.62
15	**[Zn(CH_2_SiMe_3_)(*R*,*R*)-bpzmm)]_2_^j^**, [46]	50	3.5	0.94	73	10,500	10,600	1.08	0.77

^a^ Polymerisation conditions: (a) 90 μmol of initiator, [*rac*-LA]_0_/[Zn]_0_ = 100 and 10 mL of tetrahydrofuran as solvent. ^b^ Percentage conversion of the monomer [(weight of polymer recovered/weight of monomer) × 100]. ^c^ Theoretical *M*_n_ = (monomer/initiator) × (% conversion) × (*M*_w_ of lactide). ^d^ Determined by GPC relative to polystyrene standards in tetrahydrofuran. Experimental *M*_n_ was calculated considering Mark–Houwink’s corrections [65,66] for *M*_n_ [*M*_n_(obsd.) = 0.58 × *M*_n_(GPC)]. ^e^ *P*_s_ is the probability of racemic linkages between monomer units and is determined from the relative intensity in the tetrads obtained in the decoupled ^1^H NMR by *P*_s_ = 2*I*_1_/(*I*_1_ + *I*_2_), with *I*_1_ = δ 5.20–5.25 ppm (*sis*, *sii*/*iis*) and *I*_2_ = δ 5.13–5.20 ppm (*iis*/*sii*, *iii*, *isi*) [68]. ^f^ Double-feed experiment; additional injection of 100 equiv after 3.75 h. ^g^ 25 mL of toluene as solvent. ^h^ Melt *rac*-LA monomer at 125 °C in the absence of solvent. ^i^ Melt twice-sublimed *rac*-LA monomer at 125 °C in the absence of solvent. ^j^ These data have been included for comparison in the ROP with mononuclear NNN′-amidinate-based [67], and mononuclear [42] and binuclear [46] alkoxide-based scorpionate zinc alkyl analogs. ^k^ Not determined.

## Data Availability

The data presented in this study are available on request from the corresponding author.

## Supplementary Materials

Details of spectroscopy details for complexes 1 and 2, X-ray diffraction studies for 1 and experimental details for the ring-opening polymerisation of *rac*-lactide. CCDC 2092631. For ESI and crystallographic data in CIF or other electronic format, see https://www.mdpi.com/article/10.3390/polym13142356/s1.

## References

[1] Amouroux J., Siffert P., Pierre Massué J., Cavadias S., Trujillo B., Hashimoto K., Rutberg P., Dresvin S., Wang X. (2014). Carbon Dioxide: A new Material for Energy Storage. Prog. Nat. Sci. Mater. Int..

[2] (2019). Ellen MacArthur Foundation, Completing the Picture: How the Circular Economy Tackles Climate Change. https://www.ellenmacarthurfoundation.org/assets/downloads/Completing_The_Picture_How_The_Circular_Economy-_Tackles_Climate_Change_V3_26_September.pdf.

[3] Anastas P.T., Warner J.C. (1998). Green Chemistry: Theory and Practice.

[4] Liu S., Qin S., He M., Zhou D., Qin Q., Wang H. (2020). Current Applications of Poly(Lactic Acid) Composites in Tissue Engineering and Drug Delivery. Compos. Part B Eng..

[5] Grand View Research, Polylactic Acid Market Size, Share&Trends Analysis Report By End-Use (Packaging, Textile, Agriculture, Automotive&Transport, Electronics), by Region (North America, APAC, Europe), and Segment Forecasts, 2021–2028. https://www.grandviewresearch.com/industry-analysis/polylactic-acid-pla-market.

[6] Ragauskas A.J., Williams C.K., Davison B.H., Britovsek G., Cairney J., Eckert C.A., Frederick W.J., Hallett J.P., Leak D.J., Liotta C.L. (2006). The Path Forward for Biofuels and Biomaterials. Science.

[7] Zhang X., Fevre M., Jones G.O., Waymouth R.M. (2018). Catalysis as an Enabling Science for Sustainable Polymers. Chem. Rev..

[8] Galbis J.A., García-Martín M.D.G., de Paz M.V., Galbis E. (2016). Synthetic Polymers from Sugar-Based Monomers. Chem. Rev..

[9] Guillaume S.M., Kirillov E., Sarazin Y., Carpentier J.-F. (2015). Beyond Stereoselectivity, Switchable Catalysis: Some of the Last Frontier Challenges in Ring-Opening Polymerization of Cyclic Esters. Chem. Eur. J..

[10] Cameron D.J.A., Shaver M.P. (2011). Aliphatic Polyester Polymer Stars: Synthesis, Properties and Applications in Biomedicine and Nanotechnology. Chem. Soc. Rev..

[11] Di Lorenzo M.L., Androsch R., Abdel-Rahman M.A., Biernesser A.B. (2018). Synthesis, Structure and Properties of Poly(Lactic Acid).

[12] Jiménez A., Peltzer M., Ruseckaite R. (2015). Poly(Lactic Acid) Science and Technology: Processing, Properties, Additives and Applications.

[13] Auras R. (2010). Poly(Lactic Acid) Synthesis, Structures, Properties, Processing, and Applications.

[14] Dubois P., Coulembier O., Raquez J.-M. (2009). Handbook of Ring-Opening Polymerization.

[15] Institute for Bioplastics and Biocomposites (2020). Biopolymers-Facts and Statistics.

[16] Qi J., Zhang Y., Liu X., Zhang Q., Xiong C. (2020). Preparation and Properties of A Biodegradable Poly(Lactide-Co-Glycolide)/Poly(Trimethylene Carbonate) Porous Composite Scaffold for Bone Tissue Engineering. New J. Chem..

[17] Pêgo A.P., Siebum B., Van Luyn M.J.A., Gallego y Van Seijen X.J., Poot A.A., Grijpma D.W., Feijen J. (2003). Preparation of Degradable Porous Structures Based on 1,3-Trimethylene Carbonate and D,L-Lactide (Co)Polymers for Heart Tissue Engineering. Tissue Eng..

[18] Tyler B., Gullotti D., Mangraviti A., Utsuki T., Brem H. (2016). Polylactic Acid (PLA) Controlled Delivery Carriers for Biomedical Applications. Adv. Drug Deliv. Rev..

[19] Bi H., Feng T., Li B., Han Y. (2020). In Vitro and In Vivo Comparison Study of Electrospun PLA and PLA/PVA/SA Fiber Membranes for Wound Healing. Polymers.

[20] Wu F., Misra M., Mohanty A.K. (2021). Challenges and New Opportunities on Barrier Performance of Biodegradable Polymers for Sustainable Packaging. Prog. Polym. Sci..

[21] Darensbourg D.J., Choi W., Richers C.P. (2007). Ring-Opening Polymerization of Cyclic Monomers by Biocompatible Metal Complexes. Production of Poly(Lactide), Polycarbonates, and Their Copolymers. Macromolecules.

[22] Liu N., Liu D., Liu B., Zhang H., Cui D. (2021). Stereoselective Polymerization of *rac*-Lactide Catalyzed by Zwitterionic Calcium Complexes. Polym. Chem..

[23] Bhattacharjee J., Harinath A., Sarkar A., Panda T.K. (2020). Alkaline Earth Metal-Mediated Highly Iso-selective Ring-Opening Polymerization of *rac*-Lactide. Chem. Asian J..

[24] Devaine-Pressing K., Oldenburg F.J., Menzel J.P., Springer M., Dawe L.N., Kozak C.M. (2020). Lithium, Sodium, Potassium and Calcium Amine-Bis(Phenolate) Complexes in the Ring-Opening Polymerization of *rac*-Lactide. Dalton Trans..

[25] Carpentier J.-F., Sarazin Y., Harder S. (2013). Alkaline-Earth Metal Complexes in Homogeneous Polymerization Catalysis. Alkaline-Earth Metal Compounds: Oddities and Applications.

[26] Devaine-Pressing K., Lehr J.H., Pratt M.E., Dawe L.N., Sarjeant A.A., Kozak C.M. (2015). Magnesium Amino-Bis(Phenolato) Complexes for the Ring-Opening Polymerization of *rac*-Lactide. Dalton Trans..

[27] Schofield A.D., Barros M.L., Cushion M.G., Schwarz A.D., Mountford P. (2009). Sodium, Magnesium and Zinc Complexes of Mono(Phenolate) Heteroscorpionate Ligands. Dalton Trans..

[28] Cowan J.A. (1995). The Biological Chemistry of Magnesium.

[29] Campbell N.A. (1993). Biology.

[30] Gruszka W., Walker L.C., Shaver M.P., Garden J.A. (2020). In Situ Versus Isolated Zinc Catalysts in the Selective Synthesis of Homo and Multi-block Polyesters. Macromolecules.

[31] Thevenon A., Romain C., Bennington Michael S., White Andrew J.P., Davidson Hannah J., Brooker S., Williams Charlotte K. (2016). Dizinc Lactide Polymerization Catalysts: Hyperactivity by Control of Ligand Conformation and Metallic Cooperativity. Angew. Chem. Int. Ed..

[32] Williams C.K., Breyfogle L.E., Choi S.K., Nam W., Young V.G., Hillmyer M.A., Tolman W.B. (2003). A Highly Active Zinc Catalyst for the Controlled Polymerization of Lactide. J. Am. Chem. Soc..

[33] Chamberlain B.M., Cheng M., Moore D.R., Ovitt T.M., Lobkovsky E.B., Coates G.W. (2001). Polymerization of Lactide with Zinc and Magnesium β-Diiminate Complexes:  Stereocontrol and Mechanism. J. Am. Chem. Soc..

[34] Ding K., Miranda M.O., Moscato-Goodpaster B., Ajellal N., Breyfogle L.E., Hermes E.D., Schaller C.P., Roe S.E., Cramer C.J., Hillmyer M.A. (2012). Roles of Monomer Binding and Alkoxide Nucleophilicity in Aluminum-Catalyzed Polymerization of ε-Caprolactone. Macromolecules.

[35] Normand M., Dorcet V., Kirillov E., Carpentier J.-F. (2013). {Phenoxy-Imine}Aluminum Versus-Indium Complexes for the Immortal ROP of Lactide: Different Stereocontrol, Different Mechanisms. Organometallics.

[36] Wei Y., Wang S., Zhou S. (2016). Aluminum Alkyl Complexes: Synthesis, Structure, and Application in ROP of Cyclic Esters. Dalton Trans..

[37] Aluthge D.C., Ahn J.M., Mehrkhodavandi P. (2015). Overcoming Aggregation in Indium Salen Catalysts for Isoselective Lactide Polymerization. Chem. Sci..

[38] Le Roux E. (2016). Recent Advances on Tailor-Made Titanium Catalysts for Biopolymer Synthesis. Coord. Chem. Rev..

[39] Bakewell C., White A.J.P., Long N.J., Williams C.K. (2014). Metal-Size Influence in Iso-Selective Lactide Polymerization. Angew. Chem. Int. Ed..

[40] Lee C., Hong C. (2014). An Overview of the Synthesis and Synthetic Mechanism of Poly (Lactic Acid). Mod. Chem. Appl..

[41] Honrado M., Sobrino S., Fernández-Baeza J., Sánchez-Barba L.F., Garcés A., Lara-Sánchez A., Rodríguez A.M. (2019). Synthesis of an Enantiopure Scorpionate Ligand by a Nucleophilic Addition to a Ketenimine and a Zinc Initiator for the Isoselective ROP of *rac*-Lactide. Chem. Commun..

[42] Otero A., Fernandez-Baeza J., Sanchez-Barba L.F., Sobrino S., Garces A., Lara-Sanchez A., Rodriguez A.M. (2017). Mono- and Binuclear Chiral N,N,O-Scorpionate Zinc Alkyls as Efficient Initiators for the ROP of *rac*-Lactide. Dalton Trans..

[43] Honrado M., Otero A., Fernández-Baeza J., Sánchez-Barba L.F., Garcés A., Lara-Sánchez A., Rodríguez A.M. (2016). Synthesis and Dynamic Behavior of Chiral NNO-Scorpionate Zinc Initiators for the Ring-Opening Polymerization of Cyclic Esters. Eur. J. Inorg. Chem..

[44] Honrado M., Otero A., Fernández-Baeza J., Sánchez-Barba L.F., Garcés A., Lara-Sánchez A., Rodríguez A.M. (2016). Copolymerization of Cyclic Esters Controlled by Chiral NNO-Scorpionate Zinc Initiators. Organometallics.

[45] Honrado M., Otero A., Fernández-Baeza J., Sánchez-Barba L.F., Garcés A., Lara-Sánchez A., Martínez-Ferrer J., Sobrino S., Rodríguez A.M. (2015). New Racemic and Single Enantiopure Hybrid Scorpionate/Cyclopentadienyl Magnesium and Zinc Initiators for the Stereoselective ROP of Lactides. Organometallics.

[46] Otero A., Fernández-Baeza J., Sánchez-Barba L.F., Tejeda J., Honrado M., Garcés A., Lara-Sánchez A., Rodríguez A.M. (2012). Chiral N,N,O-Scorpionate Zinc Alkyls as Effective and Stereoselective Initiators for the Living ROP of Lactides. Organometallics.

[47] Gao J., Zhu D., Zhang W., Solan G.A., Ma Y., Sun W.-H. (2019). Recent Progress in the Application of Group 1, 2&13 Metal Complexes as Catalysts for the Ring Opening Polymerization of Cyclic Esters. Inorg. Chem. Front..

[48] Garcés A., Sánchez-Barba L.F., Fernández-Baeza J., Otero A., Fernández I., Lara-Sánchez A., Rodríguez A.M. (2018). Organo-Aluminum and Zinc Acetamidinates: Preparation, Coordination Ability, and Ring-Opening Polymerization Processes of Cyclic Esters. Inorg. Chem..

[49] Otero A., Fernández-Baeza J., Lara-Sánchez A., Sánchez-Barba L.F. (2013). Metal Complexes with Heteroscorpionate Ligands Based on the Bis(Pyrazol-1-yl)Methane Moiety: Catalytic Chemistry. Coord. Chem. Rev..

[50] Sanchez-Barba L.F., Alonso-Moreno C., Garcés A., Fajardo M., Fernandez-Baeza J., Otero A., Lara-Sanchez A., Rodriguez A.M., Lopez-Solera I. (2009). Synthesis, Structures and Ring-Opening Polymerization Studies of New Zinc Chloride and Amide Complexes Supported by Amidinate Heteroscorpionate Ligands. Dalton Trans..

[51] (2016). SAINT v8.37, Bruker-AXS.

[52] Krause L., Herbst-Irmer R., Sheldrick G.M., Stalke D., SADABS (2015). Comparison of Silver and Molybdenum Microfocus X-ray Sources for Single-Crystal Structure Determination. J. Appl. Crystallogr..

[53] Sheldrick G.M. (2014). SHELX-2014, Program for Crystal Structure Refinement.

[54] Rosen T., Goldberg I., Navarra W., Venditto V., Kol M. (2017). Divergent [{ONNN}Mg–Cl] Complexes in Highly Active and Living Lactide Polymerization. Chem. Sci..

[55] Schäfer P.M., Fuchs M., Ohligschläger A., Rittinghaus R., McKeown P., Akin E., Schmidt M., Hoffmann A., Liauw M.A., Jones M.D. (2017). Highly Active N,O Zinc Guanidine Catalysts for the Ring-Opening Polymerization of Lactide. ChemSusChem.

[56] Kremer A.B., Osten K.M., Yu I., Ebrahimi T., Aluthge D.C., Mehrkhodavandi P. (2016). Dinucleating Ligand Platforms Supporting Indium and Zinc Catalysts for Cyclic Ester Polymerization. Inorg. Chem..

[57] Tschan M.J.L., Guo J., Raman S.K., Brule E., Roisnel T., Rager M.-N., Legay R., Durieux G., Rigaud B., Thomas C.M. (2014). Zinc and Cobalt Complexes Based on Tripodal Ligands: Synthesis, Structure and Reactivity toward Lactide. Dalton Trans..

[58] Navarro M., Sánchez-Barba L.F., Garcés A., Fernández-Baeza J., Fernández I., Lara-Sánchez A., Rodríguez A.M. (2020). Bimetallic Scorpionate-Based Helical Organoaluminum Complexes For Efficient Carbon Dioxide Fixation into a Variety of Cyclic Carbonates. Cat. Sci. Tech..

[59] Garcés A., Sánchez-Barba L.F., Fernández-Baeza J., Otero A., Lara-Sánchez A., Rodríguez A.M. (2017). Studies on Multinuclear Magnesium tert-Butyl Heteroscorpionates: Synthesis, Coordination Ability, and Heteroselective Ring-Opening Polymerization of *rac*-Lactide. Organometallics.

[60] Gong Y., Ma H. (2019). High Performance Benzoimidazolyl-Based Aminophenolate Zinc Complexes for Isoselective Polymerization of *rac*-Lactide. Chem. Commun..

[61] Fang C., Ma H. (2019). Ring-Opening Polymerization of *rac*-Lactide, Copolymerization of *rac*-Lactide and ε-Caprolactone by Zinc Complexes Bearing Pyridyl-Based Tridentate Amino-Phenolate Ligands. Eur. Polym. J..

[62] Yang Y., Wang H., Ma H. (2015). Stereoselective Polymerization of *rac*-Lactide Catalyzed by Zinc Complexes with Tetradentate Aminophenolate Ligands in Different Coordination Patterns: Kinetics and Mechanism. Inorg. Chem..

[63] Yu X.-F., Zhang C., Wang Z.-X. (2013). Rapid and Controlled Polymerization of *rac*-Lactide Using N,N,O-Chelate Zinc Enolate Catalysts. Organometallics.

[64] Wang Y., Zhao W., Liu D., Li S., Liu X., Cui D., Chen X. (2012). Magnesium and Zinc Complexes Supported by N,O-Bidentate Pyridyl Functionalized Alkoxy Ligands: Synthesis and Immortal ROP of ε-CL and l-LA. Organometallics.

[65] Inoue S. (2000). Immortal Polymerization: The Outset, Development, and Application. J. Polym. Sci. Part A Polym. Chem..

[66] Baran J., Duda A., Kowalski A., Szymanski R., Penczek S. (1997). Intermolecular Chain Transfer to Polymer with Chain Scission: General Treatment and Determination of kp/ktr in L,L-Lactide Polymerization. Macromol. Rapid Commun..

[67] Alonso-Moreno C., Garcés A., Sánchez-Barba L.F., Fajardo M., Fernández-Baeza J., Otero A., Lara-Sánchez A., Antiñolo A., Broomfield L., López-Solera M.I. (2008). Discrete Heteroscorpionate Lithium and Zinc Alkyl Complexes. Synthesis, Structural Studies, and ROP of Cyclic Esters. Organometallics.

[68] Drouin F., Oguadinma P.O., Whitehorne T.J.J., Prud’homme R.E., Schaper F. (2010). Lactide Polymerization with Chiral β-Diketiminate Zinc Complexes. Organometallics.

[69] Sánchez-Barba L.F., Hughes D.L., Humphrey S.M., Bochmann M. (2006). Ligand Transfer Reactions of Mixed-Metal Lanthanide/Magnesium Allyl Complexes with β-Diketimines:  Synthesis, Structures, and Ring-Opening Polymerization Catalysis. Organometallics.

