# Supplementary material for: Efficient Bulky Organo-Zinc Scorpionates for the Stereoselective Production of Poly(rac-lactide)s
Source: Polymers (Basel). 2021 Jul 19;13(14):2356. doi: 10.3390/polym13142356 (PMC8309543; doi:10.3390/polym13142356)
Supplement: Supplementary file 1 [file polymers-13-02356-s001.zip › polymers-1298558-supplementary.pdf]

Supporting Information for

Efficient Bulky Organo-Zinc Scorpionates for the Stereoselective Production of Poly(*rac*-lactide)s

Marta Navarro[1], Andrés Garcés[1,*], Luis F. Sánchez-Barba[1,*], Felipe de la Cruz-Martínez[2], Juan Fernández-Baeza[2], and Agustín Lara-Sánchez[2]

[1] Departamento de Biología y Geología, Física y Química Inorgánica, Universidad Rey Juan Carlos, Móstoles-28933-Madrid, Spain.

[2] Universidad de Castilla-La Mancha, Departamento de Química Inorgánica, Orgánica y Bioquímica- Centro de Innovación en Química Avanzada (ORFEO-CINQA), Campus Universitario, 13071-Ciudad Real, Spain.

Table of Contents

1) Spectroscopy details

Figures S1–S2. ^{1}H and ^{13}C-{^{1}H} NMR spectra of compounds **1** and **2** S3

2) X-ray diffraction studies for complex 1

Table S1. Crystal data and structure refinement for **1** S5

Table S2. Atomic coordinates (x 10^4) and equivalent isotropic displacement parameters (Å^{2}x 10^3) for **1**. U(eq) is defined as one third of the trace of the orthogonalized U^{ij} tensor S7

Table S3. Bond lengths [Å] and angles [°] for **1** S9

Table S4. Anisotropic displacement parameters (Å^{2}x 10^3) for **1**. The anisotropic displacement factor exponent takes the form: $-2p^2[h^2a^{*2}U^{11} + ... + 2 h k a^* b^* U^{12}]$ S13

Table S5. Hydrogen coordinates (x 10^4) and isotropic displacement parameters (Å^{2}x 10^3) for **1** S15

3) Experimental details for the ring-opening polymerization of *rac*-lactide

Typical polymerization procedures S17

Figure S3. GPC trace corresponding to a poly(*rac*-lactide) sample using **1** S18

Figure S4. Plot of monomer conversion (%) as a function of time (h) for the polymerization of *rac*-LA initiated by **1** S19

Figure S5. Selected areas of MALDI-ToF mass spectrum of poly(*rac*-lactide) synthesized by **1** S20

Figure S6. ^{1}H NMR spectrum of poly(*rac*-lactide)s prepared employing **1** showing the chain-end *termini* S21

Figures S7-8. Homodecoupled ^{1}H NMR spectra of poly(*rac*-lactide)s prepared by **1** S22

References S24

1) Spectroscopy details for complexes 1 and 2

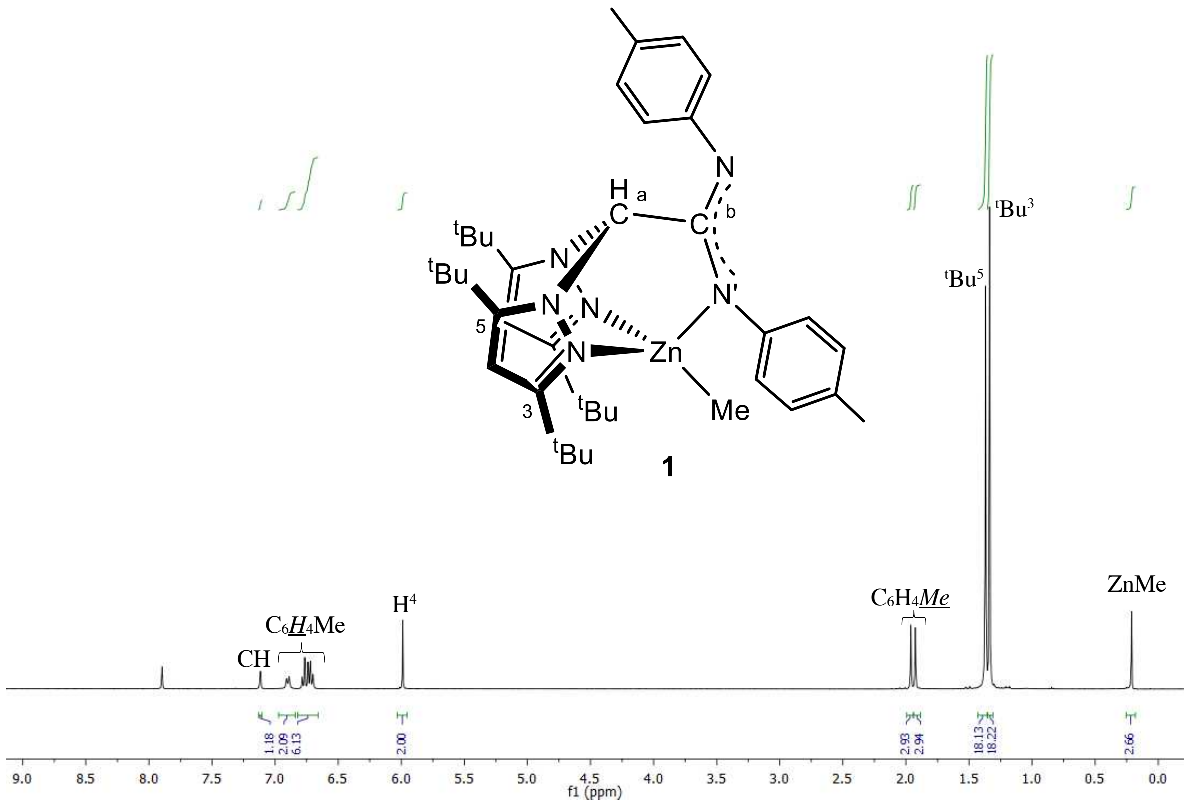

Figure S1a. ^{1}H-NMR spectrum (400 MHz, 297 K, C_6D_6) for complex [ZnMe(κ^3-phbp^{t}amd)] (**1**).

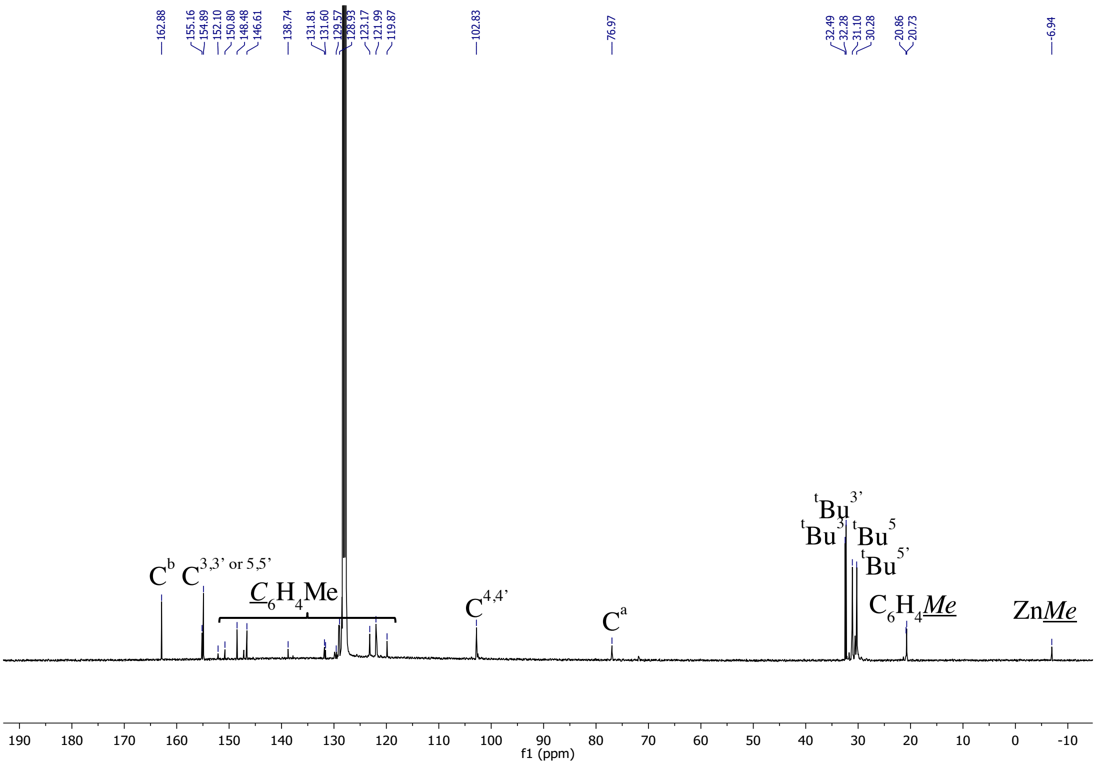

Figure S1b. ^{1}H-{^{13}C}-NMR spectrum (100 MHz, 297 K, C_6D_6) for complex [ZnMe(κ^3-phbp^{t}amd)] (**1**).

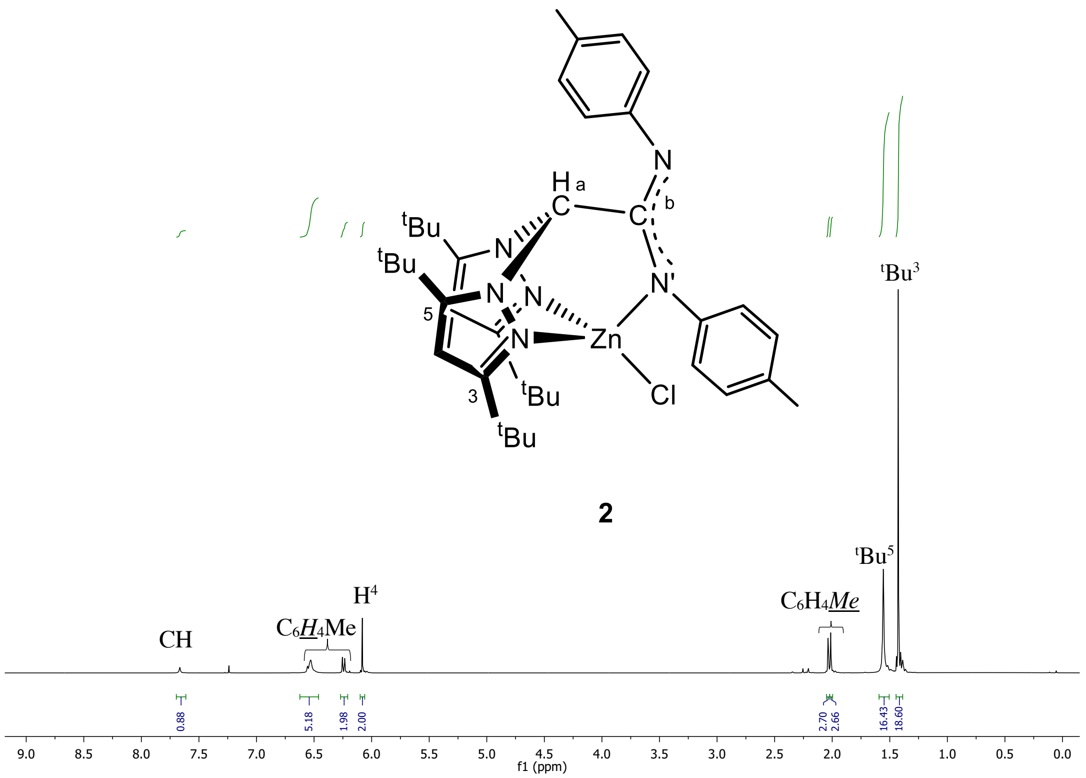

Figure S2a. ^{1}H-NMR spectrum (400 MHz, 297 K, $CDCl_3$) for complex [ZnCl(κ^3-phbp^{t}amd)] (**2**).

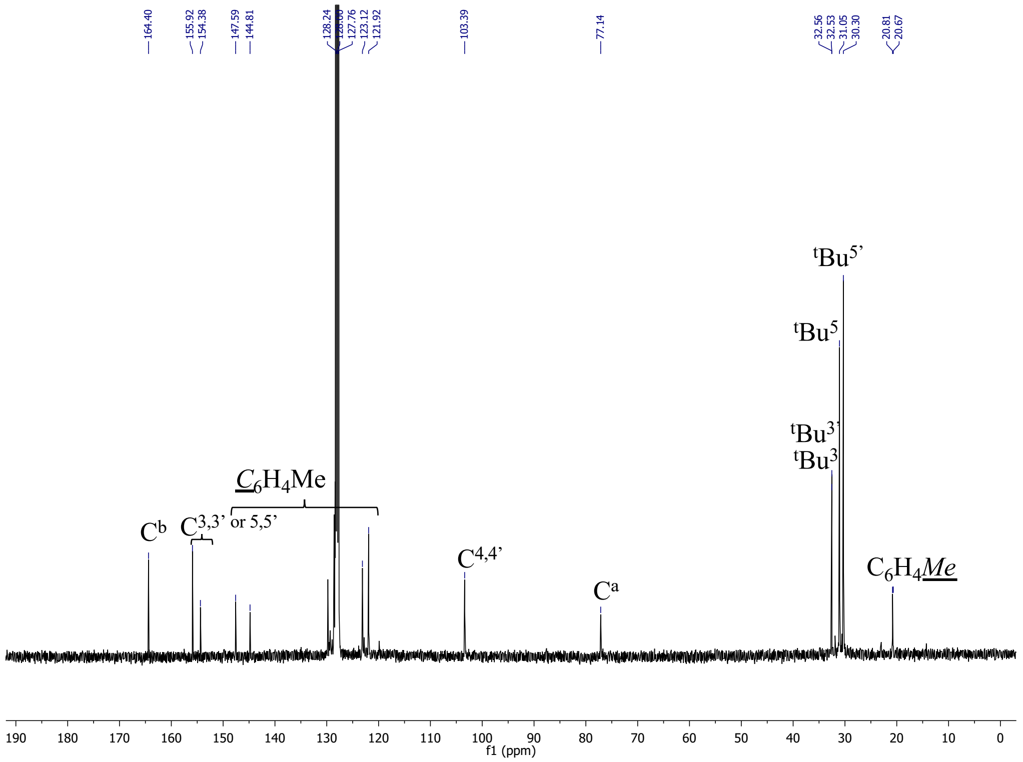

Figure S2b. ^{1}H-{^{13}C}-NMR spectrum (100 MHz, 297 K, $CDCl_3$) for complex [ZnCl(κ^3-phbp^{t}amd)] (**2**).

2) X-ray diffraction studies for complex 1.

Table S1. Crystal data and structure refinement for **1**.

Empirical formula	$C_{39} H_{56} N_6 Zn$
Formula weight	674.26
Temperature	110(2) K
Wavelength	0.71073 Å
Crystal system	Orthorhombic
Space group	P n a 2_1
a(Å)	19.8336(13)
b(Å)	11.4313(9)
c(Å)	16.4411(15)
α(°)	90
β(°)	90
γ(°)	90
Volume(Å^3)	3727.6(5)
Z	4
Density (calculated) (Mg/mm^3)	1.201
Absorption coefficient (mm^{-1})	0.693
F(000)	1448
Crystal size (mm^3)	0.200 x 0.130 x 0.090
Theta range for data collection	2.398 to 26.371°
Index ranges	-24≤h≤24, -14≤k≤14, -20≤l≤20
Reflections collected	38304
Independent reflections	7478 [R(int) = 0.1380]
Completeness to theta = 25.242°	99.9 %
Absorption correction	Semi-empirical from equivalents
Max. and min. transmission	0.745 and 0.646
Refinement method	Full-matrix least-squares on F^2
Data / restraints / parameters	7478 / 1 / 430
Goodness-of-fit on F^2	0.955

Final R indices [I>2sigma(I)]	R1 = 0.0506, wR2 = 0.0992
R indices (all data)	R1 = 0.0906, wR2 = 0.1163
Absolute structure parameter	0.007(13)
Extinction coefficient	n/a
Largest diff. peak and hole (e.Å^{-3})	0.318 and -0.384

Table S2. Atomic coordinates (x 10^4) and equivalent isotropic displacement parameters ($Å^2$x 10^3) for **1**. U(eq) is defined as one third of the trace of the orthogonalized U^{ij} tensor.

	x	y	z	U(eq)
C(1)	811(3)	-5204(5)	-2985(4)	15(1)
C(2)	1188(3)	-5594(5)	-2337(4)	18(2)
C(3)	904(3)	-6632(5)	-2056(4)	15(1)
C(4)	886(3)	-4083(5)	-3463(4)	17(1)
C(5)	219(3)	-3401(6)	-3518(5)	23(2)
C(6)	1168(3)	-4312(6)	-4318(4)	28(2)
C(7)	1398(3)	-3303(5)	-3004(4)	24(2)
C(8)	1128(3)	-7384(5)	-1355(4)	18(1)
C(9)	1904(3)	-7309(6)	-1276(4)	23(2)
C(10)	803(3)	-6910(6)	-577(4)	23(2)
C(11)	936(3)	-8671(5)	-1484(4)	22(2)
C(12)	-348(3)	-7837(5)	-4668(4)	14(1)
C(13)	-705(3)	-8859(5)	-4587(4)	16(1)
C(14)	-923(3)	-8919(5)	-3782(4)	13(1)
C(15)	37(3)	-7387(5)	-5408(4)	17(1)
C(16)	-259(4)	-6226(6)	-5732(4)	23(2)
C(17)	785(3)	-7246(6)	-5195(4)	26(2)
C(18)	-32(4)	-8282(6)	-6096(4)	33(2)
C(19)	-1326(3)	-9882(5)	-3357(4)	15(1)
C(20)	-1563(4)	-10768(6)	-3986(4)	31(2)
C(21)	-866(3)	-10492(6)	-2749(4)	25(2)
C(22)	-1938(3)	-9361(5)	-2926(5)	25(2)
C(23)	-225(3)	-6105(5)	-3683(4)	13(1)
C(24)	-900(3)	-5607(5)	-3377(4)	14(1)
C(25)	-1459(2)	-4967(4)	-2167(5)	15(1)
C(26)	-1782(3)	-5346(5)	-1469(4)	17(1)
C(27)	-2212(3)	-4614(5)	-1035(4)	20(2)
C(28)	-2337(3)	-3480(6)	-1275(4)	19(1)
C(29)	-2012(3)	-3099(5)	-1977(4)	18(2)
C(30)	-1579(3)	-3816(5)	-2418(4)	16(1)
C(31)	-2777(4)	-2660(6)	-785(4)	31(2)
C(32)	-1973(3)	-5186(5)	-3914(4)	15(1)
C(33)	-2318(3)	-4282(6)	-4300(4)	20(1)
C(34)	-3014(3)	-4251(5)	-4289(4)	21(2)

C(35)	-3399(3)	-5103(5)	-3902(4)	19(1)
C(36)	-3045(3)	-6022(5)	-3538(4)	23(2)
C(37)	-2349(3)	-6069(5)	-3537(4)	18(1)
C(38)	-4150(3)	-5025(6)	-3852(5)	31(2)
C(39)	-919(4)	-8119(6)	-1162(4)	23(2)
N(1)	365(2)	-6901(4)	-2515(3)	14(1)
N(2)	330(2)	-6043(4)	-3097(3)	13(1)
N(3)	-720(2)	-7979(4)	-3371(3)	12(1)
N(4)	-353(2)	-7324(4)	-3922(3)	13(1)
N(5)	-989(2)	-5701(4)	-2565(3)	14(1)
N(6)	-1264(2)	-5261(4)	-3985(3)	15(1)
Zn(1)	-664(1)	-7288(1)	-2166(1)	14(1)

Table S3. Bond lengths [Å] and angles [°] for **1**.

C(1)-N(2)	1.366(7)
C(1)-C(2)	1.375(8)
C(1)-C(4)	1.511(8)
C(2)-C(3)	1.392(8)
C(3)-N(1)	1.344(7)
C(3)-C(8)	1.506(9)
C(4)-C(6)	1.535(9)
C(4)-C(5)	1.538(9)
C(4)-C(7)	1.548(8)
C(8)-C(10)	1.531(9)
C(8)-C(11)	1.534(8)
C(8)-C(9)	1.547(8)
C(12)-N(4)	1.359(7)
C(12)-C(13)	1.373(8)
C(12)-C(15)	1.525(8)
C(13)-C(14)	1.393(8)
C(14)-N(3)	1.333(8)
C(14)-C(19)	1.529(8)
C(15)-C(18)	1.532(9)
C(15)-C(17)	1.534(8)
C(15)-C(16)	1.545(8)
C(19)-C(20)	1.523(8)
C(19)-C(21)	1.522(8)
C(19)-C(22)	1.527(8)
C(23)-N(2)	1.463(7)
C(23)-N(4)	1.470(7)
C(23)-C(24)	1.540(8)
C(24)-N(6)	1.295(8)
C(24)-N(5)	1.351(8)
C(25)-C(26)	1.384(9)
C(25)-C(30)	1.400(8)
C(25)-N(5)	1.414(7)
C(26)-C(27)	1.392(8)
C(27)-C(28)	1.378(9)
C(28)-C(29)	1.391(8)
C(28)-C(31)	1.513(8)
C(29)-C(30)	1.391(8)

C(32)-C(33)	1.394(8)
C(32)-C(37)	1.400(8)
C(32)-N(6)	1.413(7)
C(33)-C(34)	1.379(9)
C(34)-C(35)	1.392(9)
C(35)-C(36)	1.397(9)
C(35)-C(38)	1.495(8)
C(36)-C(37)	1.382(8)
C(39)-Zn(1)	1.970(7)
N(1)-N(2)	1.372(6)
N(1)-Zn(1)	2.167(5)
N(3)-N(4)	1.383(7)
N(3)-Zn(1)	2.135(5)
N(5)-Zn(1)	2.034(5)
N(2)-C(1)-C(2)	104.9(5)
N(2)-C(1)-C(4)	126.4(5)
C(2)-C(1)-C(4)	128.7(5)
C(1)-C(2)-C(3)	108.3(5)
N(1)-C(3)-C(2)	109.3(5)
N(1)-C(3)-C(8)	122.3(5)
C(2)-C(3)-C(8)	128.4(5)
C(1)-C(4)-C(6)	111.5(5)
C(1)-C(4)-C(5)	112.1(5)
C(6)-C(4)-C(5)	110.3(5)
C(1)-C(4)-C(7)	107.4(5)
C(6)-C(4)-C(7)	107.8(5)
C(5)-C(4)-C(7)	107.5(5)
C(3)-C(8)-C(10)	108.2(5)
C(3)-C(8)-C(11)	111.6(5)
C(10)-C(8)-C(11)	110.5(5)
C(3)-C(8)-C(9)	109.1(5)
C(10)-C(8)-C(9)	109.2(5)
C(11)-C(8)-C(9)	108.2(5)
N(4)-C(12)-C(13)	106.0(5)
N(4)-C(12)-C(15)	125.3(5)
C(13)-C(12)-C(15)	128.5(5)
C(12)-C(13)-C(14)	107.1(5)
N(3)-C(14)-C(13)	110.4(5)

N(3)-C(14)-C(19)	120.4(5)
C(13)-C(14)-C(19)	129.2(5)
C(12)-C(15)-C(18)	108.6(5)
C(12)-C(15)-C(17)	109.8(5)
C(18)-C(15)-C(17)	109.0(5)
C(12)-C(15)-C(16)	112.1(5)
C(18)-C(15)-C(16)	106.5(5)
C(17)-C(15)-C(16)	110.8(5)
C(20)-C(19)-C(21)	109.1(5)
C(20)-C(19)-C(22)	109.2(5)
C(21)-C(19)-C(22)	110.6(5)
C(20)-C(19)-C(14)	109.2(5)
C(21)-C(19)-C(14)	108.5(5)
C(22)-C(19)-C(14)	110.3(5)
N(2)-C(23)-N(4)	110.6(4)
N(2)-C(23)-C(24)	114.9(5)
N(4)-C(23)-C(24)	106.7(4)
N(6)-C(24)-N(5)	135.5(6)
N(6)-C(24)-C(23)	110.3(5)
N(5)-C(24)-C(23)	114.1(5)
C(26)-C(25)-C(30)	117.4(5)
C(26)-C(25)-N(5)	120.2(5)
C(30)-C(25)-N(5)	122.2(6)
C(25)-C(26)-C(27)	121.4(6)
C(28)-C(27)-C(26)	121.9(6)
C(27)-C(28)-C(29)	116.7(6)
C(27)-C(28)-C(31)	122.2(6)
C(29)-C(28)-C(31)	121.0(6)
C(30)-C(29)-C(28)	122.3(6)
C(29)-C(30)-C(25)	120.3(6)
C(33)-C(32)-C(37)	118.3(5)
C(33)-C(32)-N(6)	119.8(5)
C(37)-C(32)-N(6)	121.6(5)
C(34)-C(33)-C(32)	120.3(6)
C(33)-C(34)-C(35)	122.5(6)
C(34)-C(35)-C(36)	116.5(5)
C(34)-C(35)-C(38)	122.0(6)
C(36)-C(35)-C(38)	121.4(6)
C(37)-C(36)-C(35)	122.1(6)

C(36)-C(37)-C(32)	120.3(6)
C(3)-N(1)-N(2)	105.6(4)
C(3)-N(1)-Zn(1)	130.3(4)
N(2)-N(1)-Zn(1)	106.4(3)
C(1)-N(2)-N(1)	111.8(5)
C(1)-N(2)-C(23)	130.5(5)
N(1)-N(2)-C(23)	117.6(4)
C(14)-N(3)-N(4)	105.2(5)
C(14)-N(3)-Zn(1)	141.7(4)
N(4)-N(3)-Zn(1)	112.4(4)
C(12)-N(4)-N(3)	111.2(5)
C(12)-N(4)-C(23)	130.4(5)
N(3)-N(4)-C(23)	115.4(5)
C(24)-N(5)-C(25)	119.8(5)
C(24)-N(5)-Zn(1)	110.3(4)
C(25)-N(5)-Zn(1)	126.0(4)
C(24)-N(6)-C(32)	120.6(5)
C(39)-Zn(1)-N(5)	128.2(2)
C(39)-Zn(1)-N(3)	125.8(2)
N(5)-Zn(1)-N(3)	90.8(2)
C(39)-Zn(1)-N(1)	124.2(2)
N(5)-Zn(1)-N(1)	91.76(18)
N(3)-Zn(1)-N(1)	83.00(18)

Symmetry transformations used to generate equivalent atoms:

Table S4. Anisotropic displacement parameters (Å^{2}x 10^3) for **1**. The anisotropic displacement factor exponent takes the form: -2p^2[h^{2}a^{*2}U^{11} + ... + 2 h k a* b* U^{12}]

	U^{11}	U^{22}	U^{33}	U^{23}	U^{13}	U^{12}
C(1)	17(3)	11(3)	17(3)	1(3)	4(3)	0(2)
C(2)	13(3)	20(3)	20(4)	3(3)	-3(2)	1(2)
C(3)	14(3)	17(3)	13(4)	-5(3)	2(3)	3(2)
C(4)	17(3)	17(3)	18(4)	2(3)	-3(3)	-2(3)
C(5)	22(4)	17(4)	29(4)	4(3)	-5(3)	0(3)
C(6)	30(4)	28(4)	25(4)	5(3)	5(3)	-6(3)
C(7)	23(4)	22(3)	26(4)	4(3)	-6(3)	-8(3)
C(8)	15(3)	17(3)	22(3)	5(3)	-5(3)	4(3)
C(9)	19(3)	27(4)	23(4)	5(3)	-7(3)	7(3)
C(10)	26(4)	22(3)	21(4)	4(3)	-1(3)	7(3)
C(11)	27(3)	16(3)	22(4)	4(3)	-5(3)	3(3)
C(12)	12(3)	17(3)	11(3)	2(3)	-1(2)	8(3)
C(13)	18(3)	14(3)	15(3)	-2(2)	0(3)	2(3)
C(14)	6(3)	16(3)	18(4)	2(3)	-1(2)	1(2)
C(15)	22(3)	17(3)	13(3)	3(3)	4(3)	-1(3)
C(16)	35(4)	20(4)	15(4)	1(3)	3(3)	0(3)
C(17)	17(3)	29(4)	31(4)	4(3)	11(3)	3(3)
C(18)	52(5)	31(4)	14(4)	-9(3)	12(3)	-4(4)
C(19)	14(3)	17(3)	15(3)	3(3)	1(3)	-1(3)
C(20)	41(4)	29(4)	22(4)	-8(3)	3(3)	-15(3)
C(21)	23(3)	21(3)	31(4)	7(3)	-2(3)	-1(3)
C(22)	13(3)	21(3)	41(5)	0(3)	4(3)	-3(3)
C(23)	13(3)	13(3)	14(3)	-1(3)	0(2)	-2(2)
C(24)	14(3)	10(3)	19(4)	-1(3)	2(3)	-4(2)
C(25)	9(2)	21(3)	14(3)	-4(4)	3(3)	0(2)
C(26)	21(3)	18(3)	14(4)	2(3)	-1(3)	-3(3)
C(27)	17(3)	25(4)	17(4)	1(3)	7(3)	-2(3)
C(28)	10(3)	30(4)	18(4)	-6(3)	3(3)	0(3)
C(29)	19(3)	17(3)	17(4)	1(2)	-2(3)	0(2)
C(30)	14(3)	18(3)	18(4)	-1(2)	3(2)	-3(3)
C(31)	33(4)	30(4)	30(4)	-11(3)	7(3)	8(3)
C(32)	13(3)	17(3)	14(3)	-6(3)	0(3)	1(3)
C(33)	18(3)	25(3)	15(3)	2(3)	-1(3)	2(3)
C(34)	21(3)	20(3)	22(4)	1(3)	-3(3)	6(3)

C(35)	17(3)	23(3)	18(3)	-5(3)	-5(3)	2(3)
C(36)	14(3)	19(3)	34(4)	-7(3)	0(3)	-5(3)
C(37)	14(3)	17(3)	22(4)	-3(3)	1(3)	3(3)
C(38)	21(4)	29(4)	41(5)	-7(3)	1(3)	3(3)
C(39)	24(4)	21(4)	22(4)	4(3)	1(3)	-2(3)
N(1)	14(2)	13(2)	15(3)	3(2)	-1(2)	-2(2)
N(2)	13(3)	13(2)	13(3)	2(2)	1(2)	1(2)
N(3)	14(3)	12(3)	10(3)	2(2)	0(2)	-3(2)
N(4)	14(2)	13(2)	12(3)	-1(2)	1(2)	-2(2)
N(5)	13(3)	16(3)	13(3)	0(2)	1(2)	-1(2)
N(6)	13(3)	16(3)	17(3)	1(2)	-1(2)	2(2)
Zn(1)	16(1)	14(1)	13(1)	2(1)	1(1)	-1(1)

Table S5. Hydrogen coordinates (x 10^4) and isotropic displacement parameters (Å^{2}x 10^3) for **1**.

	x	y	z	U(eq)
H(2)	1567	-5228	-2123	21
H(5A)	-113	-3872	-3788	34
H(5B)	289	-2693	-3820	34
H(5C)	65	-3212	-2980	34
H(6A)	1548	-4830	-4281	41
H(6B)	1308	-3586	-4558	41
H(6C)	826	-4665	-4649	41
H(7A)	1233	-3145	-2467	35
H(7B)	1455	-2579	-3293	35
H(7C)	1823	-3699	-2969	35
H(9A)	2110	-7601	-1764	35
H(9B)	2049	-7770	-820	35
H(9C)	2035	-6509	-1194	35
H(10A)	946	-6117	-491	34
H(10B)	938	-7382	-123	34
H(10C)	321	-6934	-631	34
H(11A)	456	-8734	-1545	32
H(11B)	1078	-9123	-1022	32
H(11C)	1154	-8961	-1965	32
H(13)	-786	-9408	-4993	19
H(16A)	-738	-6300	-5789	35
H(16B)	-63	-6051	-6252	35
H(16C)	-158	-5606	-5358	35
H(17A)	830	-6749	-4727	38
H(17B)	1019	-6901	-5647	38
H(17C)	976	-7999	-5078	38
H(18A)	155	-9016	-5925	49
H(18B)	205	-8007	-6567	49
H(18C)	-501	-8385	-6226	49
H(20A)	-1843	-10384	-4381	46
H(20B)	-1817	-11374	-3722	46
H(20C)	-1179	-11106	-4254	46
H(21A)	-489	-10829	-3031	37
H(21B)	-1112	-11098	-2475	37

H(21C)	-706	-9934	-2358	37
H(22A)	-1792	-8781	-2543	37
H(22B)	-2178	-9969	-2646	37
H(22C)	-2231	-9005	-3320	37
H(23)	-92	-5668	-4170	16
H(26)	-1711	-6106	-1286	21
H(27)	-2422	-4899	-569	24
H(29)	-2087	-2339	-2157	21
H(30)	-1368	-3529	-2883	20
H(31A)	-3081	-3109	-455	46
H(31B)	-3031	-2170	-1147	46
H(31C)	-2499	-2182	-442	46
H(33)	-2079	-3696	-4567	24
H(34)	-3233	-3637	-4549	25
H(36)	-3286	-6621	-3289	27
H(37)	-2130	-6690	-3285	21
H(38A)	-4274	-4461	-3446	46
H(38B)	-4331	-5776	-3709	46
H(38C)	-4327	-4788	-4370	46
H(39A)	-655	-8819	-1112	34
H(39B)	-1389	-8320	-1184	34
H(39C)	-839	-7623	-701	34

3) Experimental details for the synthesis of poly(*rac*-lactide) 3

Typical polymerization procedures

Polymerizations of *rac*-lactide (LA) were performed on a Schlenk line in a flame-dried Schlenk tube equipped with a magnetic stirrer. The Schlenk tubes were charged in a glovebox with the required amount of LA and initiator, separately, and then attached to the vacuum line. The initiator and LA were dissolved in the appropriate amount of solvent and temperature equilibration was ensured in both Schlenk tubes by stirring the solutions for 15 min in a bath. Next, the appropriate amount of initiator was added by using a syringe and polymerization times were measured from that point. Polymerizations were stopped by injecting a solution of acetic acid in water (0.35 M). Polymers were precipitated in methanol, filtered off, redissolved and reprecipitated in methanol, and dried in vacuo to a constant weight.

==== Shimadzu LCsolution Analysis Report ====

Acquired by	: Admin
Sample Name	:lf23
Sample ID	:12
Vail #	:
Injection Volume	: 20 uL
Data File Name	: lf23.lcd
Method File Name	:
Batch File Name	:
Report File Name	: Default.lcr
Data Acquired	: 12/12/2020 15:52:15
Data Processed	: 12/12/2020 15:58:26

<Chromatogram>

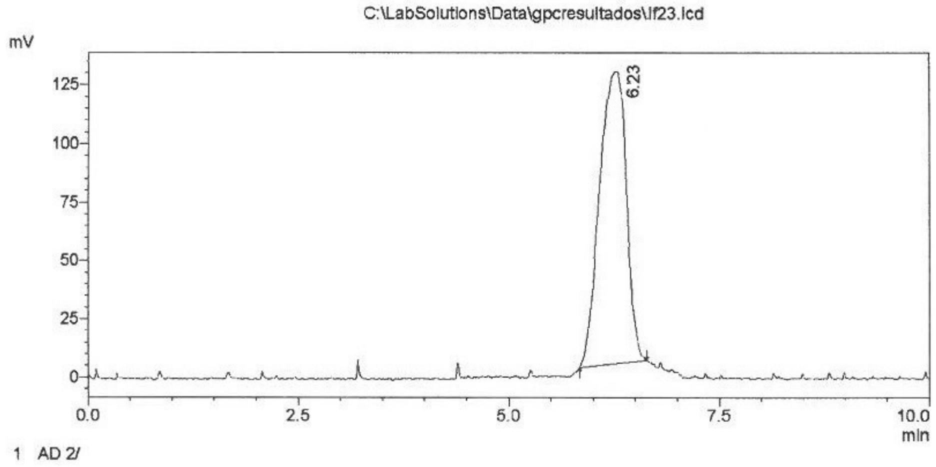

GPC Results

Peak#:1 (AD2)

[Average Molecular Weight]

Number Average Molecular Weight(Mn)	18073
Weight Average Molecular Weight(Mw)	16139
Viscosity Average Molecular Weight(Mv)	0
Mw/Mn	1.119

Figure S3. GPC trace corresponding to a poly(*rac*-lactide) **3** prepared from catalyst [ZnMe(κ^3-phbp^{t}amd)] **1** (Table 2, entry 3).

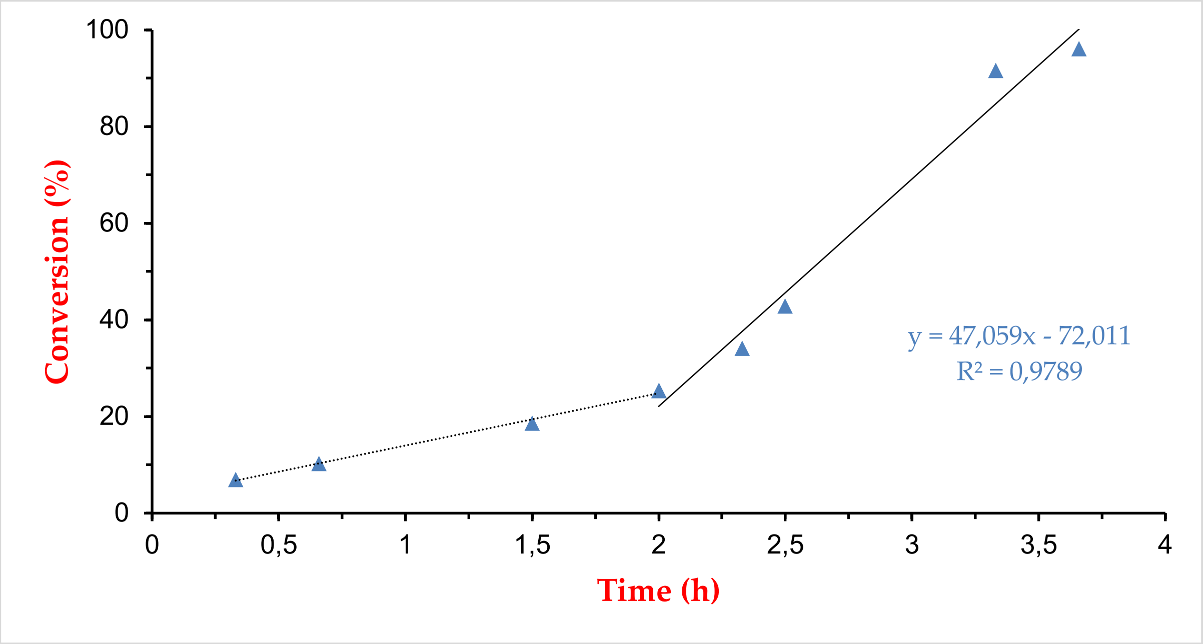

Figure S4. Plot of monomer conversion (%) as a function of time (h) for the polymerization of *rac*-LA initiated by [ZnMe(κ^3-phbp^{t}amd)] **1**; $[rac\text{-LA}]_0/[\text{Zn}]_0 = 100$, tetrahydrofuran, 50 ºC. Induction period of 2 h.[1] Reaction completed after 3.75 h. (Table 2, entries 1–4, and additional experimental data).

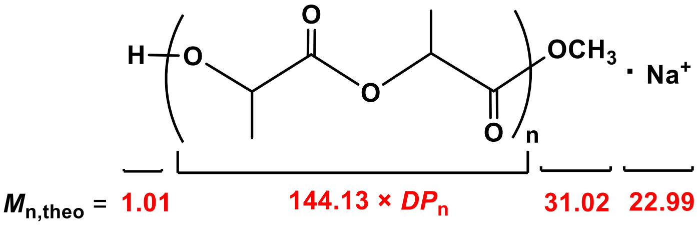

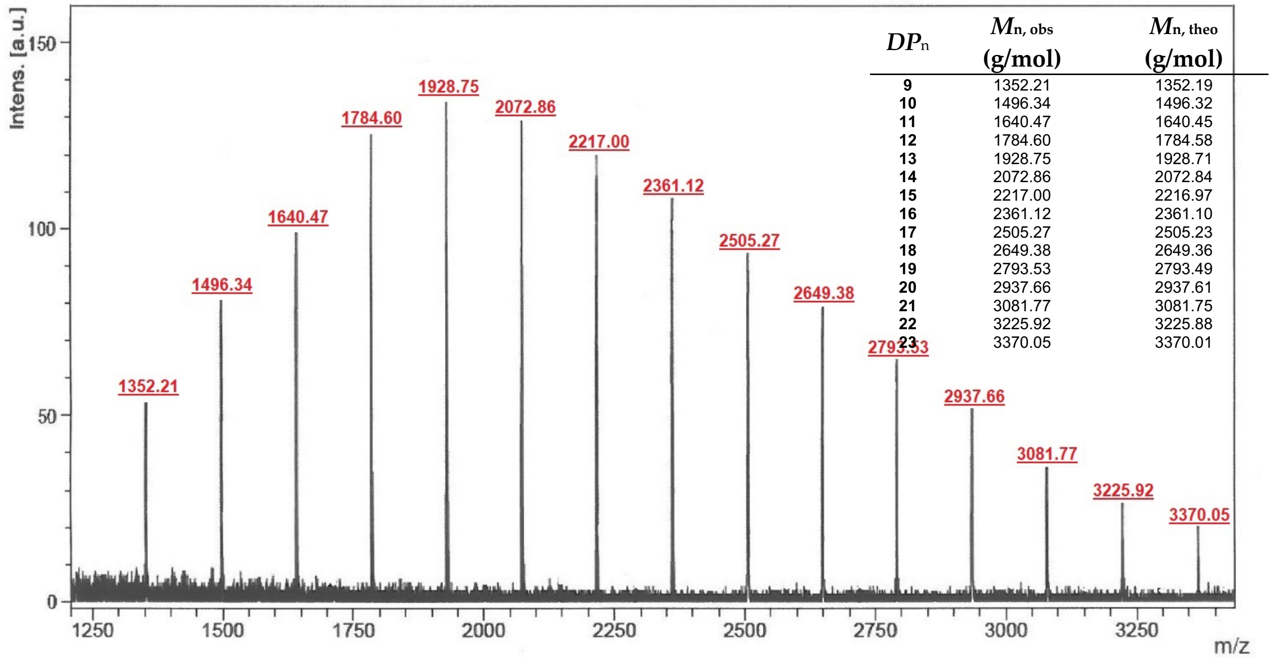

DP_n	$M_{n,\,obs}$ (g/mol)	$M_{n,\,theo}$ (g/mol)
**9**	1352.21	1352.19
**10**	1496.34	1496.32
**11**	1640.47	1640.45
**12**	1784.60	1784.58
**13**	1928.75	1928.71
**14**	2072.86	2072.84
**15**	2217.00	2216.97
**16**	2361.12	2361.10
**17**	2505.27	2505.23
**18**	2649.38	2649.36
**19**	2793.53	2793.49
**20**	2937.66	2937.61
**21**	3081.77	3081.75
**22**	3225.92	3225.88
**23**	3370.05	3370.01

Figure S5. Selected area of the MALDI-ToF mass spectrum of a PLA sample obtained on using [ZnMe(κ^3-phbp^{t}amd)] **1** with $[Zn]_0:[rac\text{-}LA]_0 = 30$, 77% conversion, after quenching in methanol as solvent. Theoretical molecular weights calculated according to the equation: $M_n = (DP_n \times M_{wLA}) + M_{w\,HOCH3} + M_{wNa}$, where DP_n is the degree of polymerization, $M_{wLA} = 144.13$ g·mol^{-1}, $M_{wHOCH3} = 32.03$ g·mol^{-1} and $M_{wNa} = 22.99$ g·mol^{-1}.

The distribution in the spectrum indicates the existence of a single family of polymer chains capped by –CH(CH_3)OH and CH_3O–C–OC(O)– *termini*, corresponding to oligomers of formula H(OCHMeCO)$_{2n}$(C-OCH_3)·Na^+ (n = 9 to 23) with consecutive peaks separated by increments of 144.13 Da. Moreover, neither intermolecular ester-exchange (transesterification) reactions nor cyclic oligomers were detected.

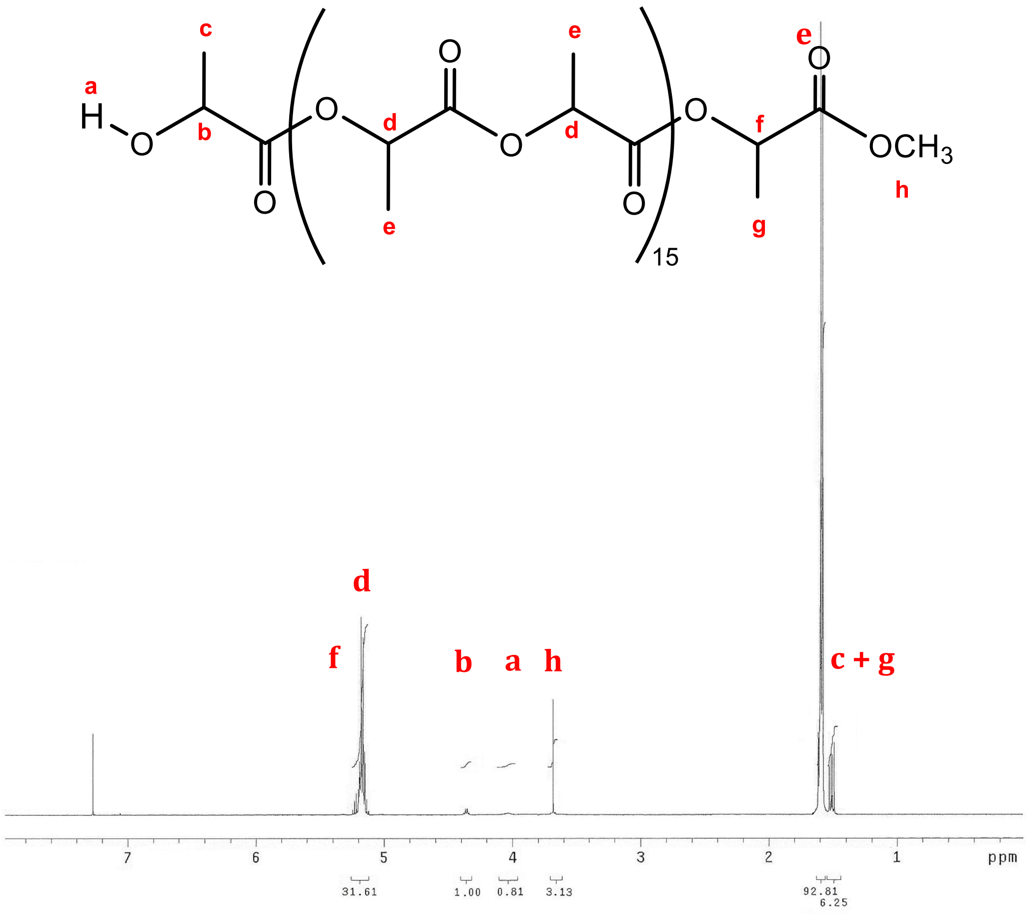

Figure S6. ^{1}H NMR spectrum (400 MHz, 298 K, $CDCl_3$) of PLA prepared by the polymerization of *rac*-LA initiated by [ZnMe(κ^3-phbp^{t}amd)] (**1**) at 64% of conversion after quenching in methanol as solvent ($[Zn]_0$:[*rac*-LA]$_0$ = 1:25, tetrahydrofuran, 50 ºC, 3 h), showing all resonances and assignments, including the chain *termini*.

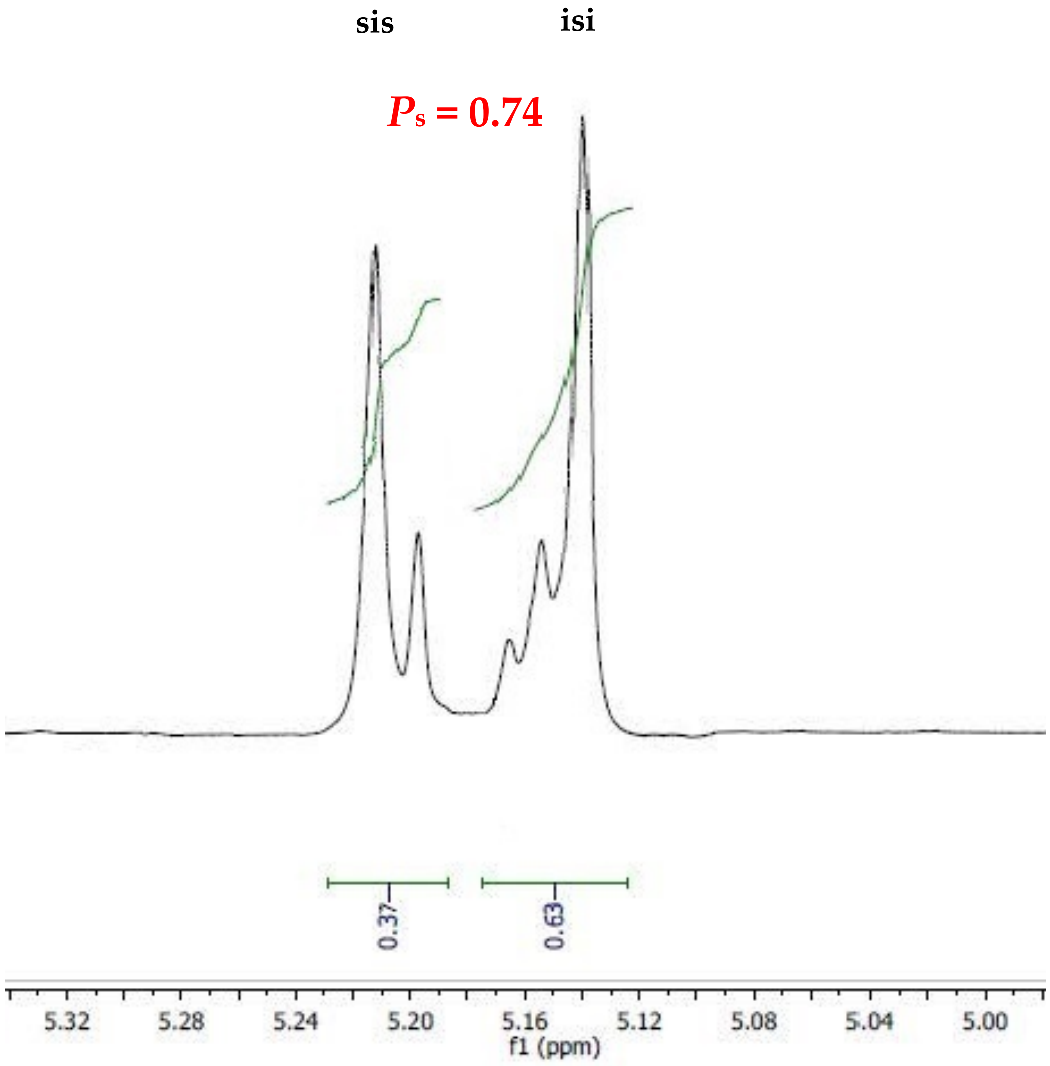

Figure S7. ^{1}H NMR spectra (400 MHz, 298 K, $CDCl_3$) of the homodecoupled C*H* resonance of poly(*rac*-lactide) prepared employing [ZnMe(κ^3-phbp^{t}amd)] **1** in tetrahydrofuran at 50 ºC for 3.75 h (Table 2 entry 4). The tacticity of the polymer was assigned using the methine signals with homonuclear decoupling, as described by Hillmyer and co-workers.[2]

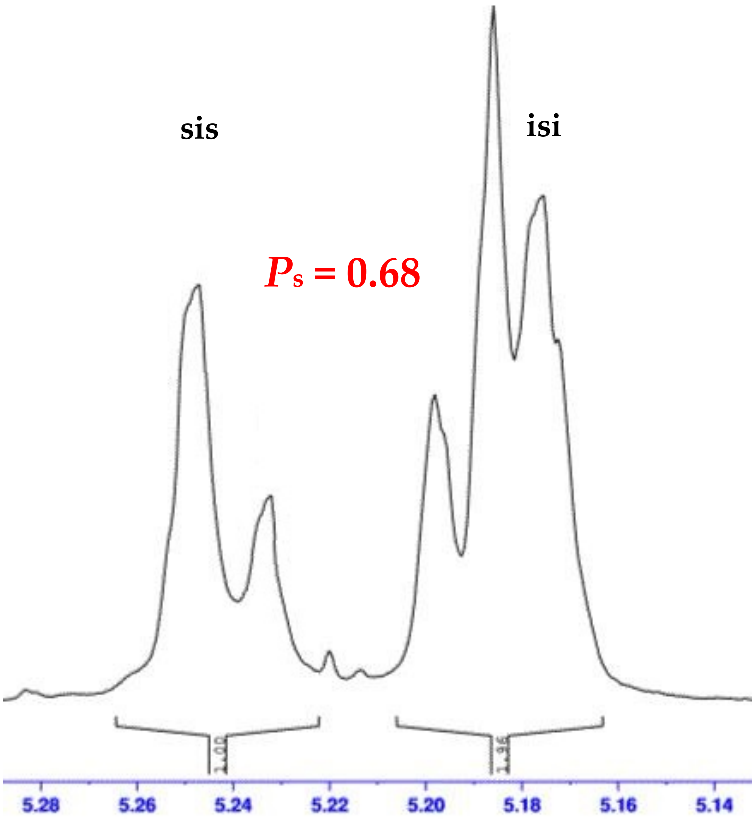

Figure S8. ^{1}H NMR spectra (400 MHz, 298 K, $CDCl_3$) of the homodecoupled C*H* resonance of poly(*rac*-lactide) prepared employing [ZnMe(κ^3-phbp^{t}amd)] (**1**) under bulk conditions (neat *rac*-LA at 125 ºC) in 1 min (Table 2 entry 10). The tacticity of the polymer was assigned using the methine signals with homonuclear decoupling, as described by Hillmyer and co-workers.[2]

References

1. Thevenon, A.; Romain, C.; Bennington, M. S.; White, A. J. P.; Davidson, H. J.; Brooker, S.; Williams, C. K. Dizinc lactide polymerization catalysts: Hyperactivity by control of ligand conformation and metallic cooperativity. *Angew. Chem., Int. Ed.* **2016**, *55*, 8680−8685.

2. Zell, M. T.; Padden, B. E.; Paterick, A. J.; Thakur, K. A. M.; Kean, R. T.; Hillmyer, M. A.; Munson, E. J. Unambiguous Determination of the ^{13}C and ^{1}H NMR Stereosequence Assignments of Polylactide Using High-Resolution Solution NMR Spectroscopy. *Macromolecules* 2002, **35**, 7700–7707.
